# Connecting livestock disease dynamics to human learning and biosecurity decisions

**DOI:** 10.3389/fvets.2022.1067364

**Published:** 2023-01-20

**Authors:** Gabriela Bucini, Eric M. Clark, Scott C. Merrill, Ollin Langle-Chimal, Asim Zia, Christopher Koliba, Nick Cheney, Serge Wiltshire, Luke Trinity, Julia M. Smith

**Affiliations:** ^1^Department of Plant and Soil Science, University of Vermont, Burlington, VT, United States; ^2^Social-Ecological Gaming and Simulation Lab, University of Vermont, Burlington, VT, United States; ^3^Department of Computer Science, University of Vermont, Burlington, VT, United States; ^4^Department of Community Development and Applied Economics, University of Vermont, Burlington, VT, United States; ^5^Food Systems Research Center, University of Vermont, Burlington, VT, United States; ^6^Computational Biology Research and Analytics Lab, University of Victoria, Victoria, BC, Canada; ^7^Department of Animal and Veterinary Sciences, University of Vermont, Burlington, VT, United States

**Keywords:** biosecurity, human behavior (activities), risk attitude, swine production systems, agent-based modeling, porcine epidemic diarrhea virus (PEDv)

## Abstract

The acceleration of animal disease spread worldwide due to increased animal, feed, and human movement has driven a growing body of epidemiological research as well as a deeper interest in human behavioral studies aimed at understanding their interconnectedness. Biosecurity measures can reduce the risk of infection, but human risk tolerance can hinder biosecurity investments and compliance. Humans may learn from hardship and become more risk averse, but sometimes they instead become more risk tolerant because they forget negative experiences happened in the past or because they come to believe they are immune. We represent the complexity of the hog production system with disease threats, human decision making, and human risk attitude using an agent-based model. Our objective is to explore the role of risk tolerant behaviors and the consequences of delayed biosecurity investments. We set up experiment with Monte Carlo simulations of scenarios designed with different risk tolerance amongst the swine producers and we derive distributions and trends of biosecurity and porcine epidemic diarrhea virus (PEDv) incidence emerging in the system. The output data allowed us to examine interactions between modes of risk tolerance and timings of biosecurity response discussing consequences for disease protection in the production system. The results show that hasty and delayed biosecurity responses or slow shifts toward a biosecure culture do not guarantee control of contamination when the disease has already spread in the system. In an effort to support effective disease prevention, our model results can inform policy making to move toward more resilient and healthy production systems. The modeled dynamics of risk attitude have also the potential to improve communication strategies for nudging and establishing risk averse behaviors thereby equipping the production system in case of foreign disease incursions.

## Introduction

Worldwide, millions of farm animals are exposed to infectious diseases on a regular basis. Humans have a pivotal part to play to protect animals and reduce the risk of infection. The incursion of a foreign animal disease (FAD) into a country previously free of the disease has the potential for devastating consequences including not only direct death of animals but also disease-response actions such as animal depopulation or movement control zones, market import/export restrictions and human health threats. For example, the spread of African Swine Fever (ASF) to countries near the United States has raised serious concerns in the United States pork industry because of the virus's high contagion rate and up to 100% pig mortality (1–3). The incursion of ASF into the United States would result in disastrous economic losses and destabilization, and indirect effects even in the human healthcare sector (4). Knowledge and awareness of the disease's pathology and geographic spread have motivated the United States pork industry as well as governmental agencies to lay out strategic plans and trainings for disease prevention and response (3, 5). It is indeed understood that humans *can* control the risk of disease spread but, given the existing variability in human risk perception and behavior, what does it take for them to control the disease?

Biosecurity protocols targeting bio-exclusion are specifically designed to guide human decisions on agricultural practices and protect the production system from animal disease incursions. Important reasons for continuous support and promotion of biosecurity practices are that technical skills need to be kept up to date and also that human dimensions such as personality and risk attitudes influence decisions on whether to invest in and comply with biosecurity (6–12). For all the successes in livestock disease prevention, many problems related to biosecurity investments and compliance persist (7, 13–15). Some of these problems may be challenging to explore in the real world because of practical obstacles inherent to the risk of disease spread. Modeling is therefore a key tool to explore scenarios and investigate mechanisms acting in a production system that may hinder or endorse disease control efforts. The goal of this article is to apply agent-based modeling to disease prevention science with a research focus on social dynamics that connects human risk attitudes to decisions on biosecurity. The human role is particularly interesting because it can alter outcomes expected from lab-tested biosecurity implementation.

Computational science in the form of agent-based models (ABM) is instrumental for exploring patterns of disease spread emerging in complex systems characterized by many individual decisions, dynamic interactions, stochasticity and varying environments. Epidemiological models continue to evolve thanks both to emerging analytic tools and near real-time data collection (16–22). To our knowledge, however, the majority of current epidemiological models rarely include dynamics of risk attitude, its effects on individual responses to disease presence and adaptive behaviors. An example of the importance of human behavior is given by Nicolas et al. (23) where transmission patterns of infectious diseases were clearly connected to cultural events as well as to human and animal seasonal movements. Social-ecological system studies offer insights into understanding human risk attitude, changing risk perceptions and consequent actions for disease prevention or response. Over the course of several years, we have been tackling these questions with a complementary set of research tools (24) applied to understanding the impacts of risk attitudes on the spread of porcine epidemic diarrhea virus (PEDv). In this paper, we investigate *via* a series of simulated scenarios the effect of risk tolerance on behaviors that influence disease control. We pay particular attention to the role played by timing of biosecurity interventions as producer agents learn more risk averse behaviors while disease spreads in the system.

First diagnosed in the United States in the spring of 2013, PEDv spread quickly to 13 states in < 2 months (25). The causative virus is highly infectious and pathogenic with a brief incubation period of about 2 days and ranging between 1 and 8 days (26). The virus causes vomiting, severe watery diarrhea and dehydration, and it affects pigs of all ages (27, 28). PEDv is a coronavirus (family: *coronoaviridae*) with infectivity resulting from a small infectious dose (28, 29). Among nursing piglets the infection spreads rapidly with almost a 100% death loss. Little death has been observed in weaned and older pigs, but there is morbidity, suffering and visible weight loss. Adults become immune, temporarily, after recovery. PEDv can survive in manure and in animal feed (30, 31) with cold temperature increasing its survival (32). It can be transmitted *via* both direct and indirect contact, especially through the fecal-oral route. Indirect transmission can occur *via* contaminated fomites including farm clothing (32), feed (31, 33–35), and transport trailers (36, 37). Airborne PEDv is another transmission mechanism that occurs *via* the fecal-nasal route from pig to pig or *via* aerosolized particles that move from farm to farm (28, 38). Multiple transmission routes, including with feed, made it particularly challenging to control PED with traditional biosecurity practices.

PED is among the most devastating viral diseases of swine in the United States and the world, leading to significant financial concerns for the pork industry (39–43). The large losses caused by PED can be significantly reduced with biosecurity strategies that control the virus in the different nodes of the production chain (swine production sites, feed mills, meat processing plants) as well as during the movement of animals and products (42). Producers can invest in and implement biosecurity measures with the aim to control or eradicate PED virus infection. Some examples of biosecurity used in a production facility are setting a line of separation, hot water cleaning with detergent, disinfecting, quarantine of animals and products, heating and drying vehicles, removing manure thoroughly, removing dead animals, disinfection of personnel and equipment, and keeping visitor logs (44–46). When we reflect on who is engaged in these procedures, we understand that, while disease contagion is clearly linked to the epidemiological character of a disease*, humans control transmission with their behaviors and activities*.

This study is part of a wider program that is focused on developing methods to understand underlying human-behavioral processes that influence the efficacy of biosecurity during a disease outbreak in an animal production system. This paper presents an agent-based model (ABM) which represents a swine production system, designed to capture behavioral aspects associated with decision-making in a context of animal-disease risk. Decision-making metrics were parameterized using information derived from experimental games. We illustrate the potential of the approach combining ABM and experimental games with PEDv spreading within a swine production system. We account for both epidemiological and human-related risk factors that affect biosecurity practices and consequent disease incidence. Building on previously published research and modeling efforts (47, 48), we present improvements in several aspects of the ABM that, we believe, make it more realistic and able to provide new insights. These improvements are guided by developments in experimental games that provide observations on how human decision-making affects biosecurity investments and compliance as well as on changes in risk perception in the participants (8, 10–12, 24, 49, 50). In general, higher perception of disease risk shifts behavior toward more biosecurity investments and higher biosecurity compliance (49, 51). Although it may appear self-evident that disease incidence should increase as the population comprises more risk tolerant individuals, this argument ignores potential complex dynamics arising from risk behavioral changes during a disease outbreak.

The overall objectives of this work are to: (1) explore the role of risk tolerant behaviors on system-level biosecurity and disease incidence, and (2) conduct an evaluation of the consequences of delayed biosecurity investments on disease control. We are interested in answering three questions:

- If we initialize the ABM with a risk tolerant population of swine producers rather than a risk naïve (calibrated model set up) population, how does disease incidence evolve after a breakout?
- If we model biosecurity investment at increasingly higher levels of tolerance (delayed investments), do we observe a tolerance scenario where percolations of disease in the system (pandemics) become the majority of simulation outcomes?
- How important is learning risk-aversion to counteracting initial conditions of risk tolerance?

The idea for these questions came from conversations with veterinarians in our advisory team who remembered the 2013 PEDv outbreak in the United States and observed first hand that the time it took for the system to upgrade their biosecurity caused high disease-transmission rates and production losses for about 2 years. We simulate this situation of low preparedness and delayed biosecurity response by encoding risk tolerant behaviors. The results will contribute reflections on the importance of timing biosecurity response and can inform design of biosecurity policies.

## Methods

**Figure 1:**
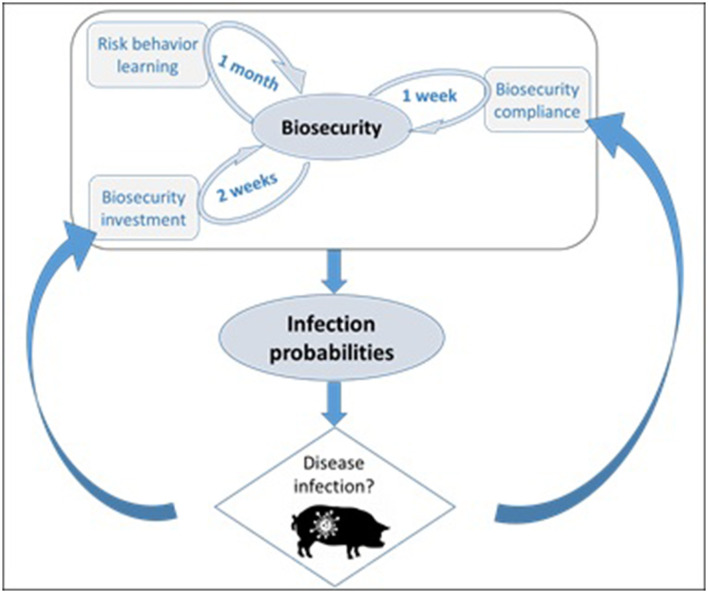
Infection probability is directly related to biosecurity in the ABM. The biosecurity level of a hog-producer agent in the model depends on its compliance, willingness of making biosecurity investments and on its risk attitude, which can change with time (learning). Disease infection feeds back in biosecurity in two ways: biosecurity investments in response to disease vicinity, and full compliance when a premises turns symptomatic. The implemented learning mechanism is agnostic and therefore independent of simulated disease conditions.

Our model links biosecurity—investment and compliance—to infection dynamics at two different scales, within networks of production premises and in individual premises (Figure 1). Note that in the paper, we use the term hog producer agent, hog producer and production premises interchangeably. The willingness of a producer agent to invest in biosecurity is based on network dynamics. Each producer is part of a network of premises overseen by a veterinarian who communicates the level of infection in the network. A producer decides whether or not to invest in biosecurity depending on the perceived risk of infection when the veterinarian sends information. Biosecurity compliance is based instead on what happens at the scale of a premises and it is set to decline with time while the premises is disease free, but it is set to the premises' maximum biosecurity capacity as soon as the premises becomes symptomatic of disease.

### Model architecture

In this section, we provide an overview of the model architecture and functioning. More details can be found in the ODD + D document in the Supplementary material. The ABM was developed in the AnyLogic software (https://www.anylogic.com) with the code written in Java (https://www.oracle.com/technetwork/java/index.html). The model architecture simulates a swine production system with agents representing production premises, feed mills and slaughter plants. The system simulated for this study mirrors the density, operation types and sizes of production units found in North Carolina with data provided by the Farm Location and Agricultural Production Simulator (FLAPS) tool, which draws from the 2012 Census of Agriculture and aerial photography (52). This is a region in which swine are intensively raised. The ABM production system has the six types of producer agents found in United States swine production chain: farrow-to-wean, wean-to-feeder, feeder-to-finish, farrow-to-finish, wean-to-finish, and feeder-to-finish. Networks are built with producer agents and service area agents (feed mills and slaughter plants) trading according to their role in the industry, and their links have a nearest neighbor structure. These networks underlie the movement of livestock and feed and encode the contact patterns between agents that the pathogens follow as the epidemic spreads.

#### Epidemiology

**Figure 2:**
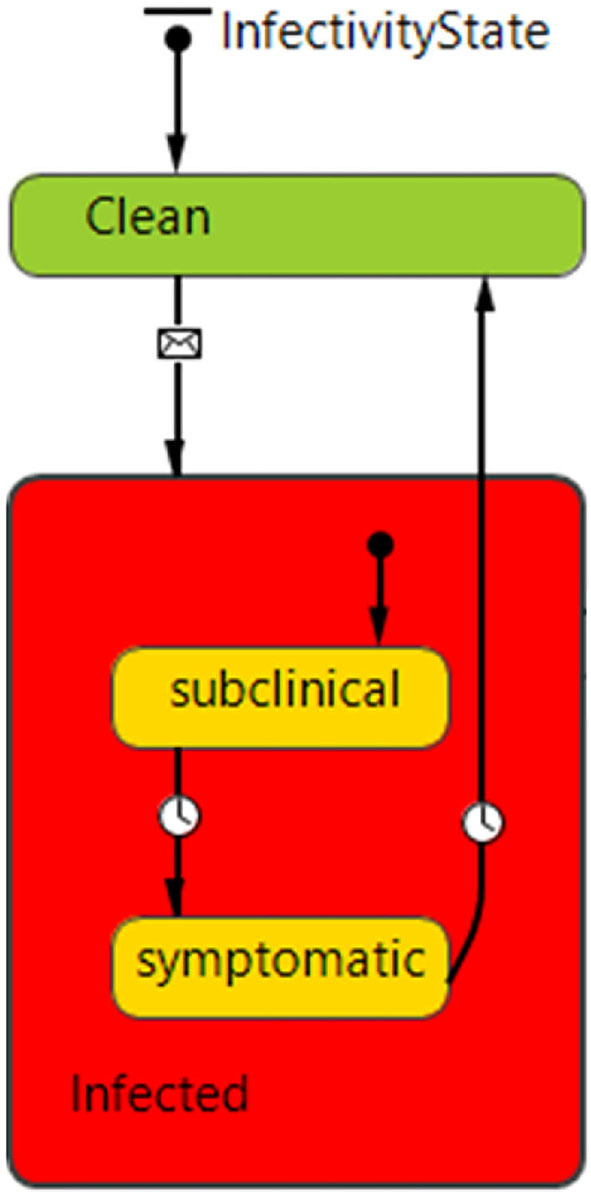
InfectivityState state chart defining disease states and transitions for hog producer agents. Each agent starts in the “Clean” state (no disease) and can transition to the infected state, which is a composite state consisting of subclinical (asymptomatic) and symptomatic sub-states. Transitions are regulated by messages and timeout conditions (2 days between subclinical and symptomatic, 50 days from Infected symptomatic to Clean—susceptible). The feed mill and slaughter plant agents can only be clean or infected.

The ABM epidemiological component simulates disease transmission *via* both direct and indirect mechanisms linked to the movement of animals and feed across networks of agents. The model does not explicitly model airborne transmission of PEDv (28). Each agent (hog producer, slaughter plant and feed mill) has a stochastic state transition model including Clean (susceptible), Infected subclinical (asymptomatic) and Infected symptomatic states (Figure 2). Disease transmission can occur during any agent interaction with probability functions dependent on three variables: agent's biosecurity, the type of network interaction and seasonality. Coefficients were estimated using expert opinion. The inclusion of a disease incubation time regulating the transition from the subclinical to the symptomatic state has been an important addition to the current ABM epidemiological component compared to previous versions (47, 48). The incubation period of infectious diseases is the time from infection to onset of symptoms. The infected host can be infectious during the incubation period and therefore the presence of this parameter is directly relevant for epidemiologic dynamics as well as for prevention and control. The disease incubation period allows us to model distinct behaviors during the asymptomatic and symptomatic states of infection. During the incubation time, a premises is modeled to operate routinely with in and out exchanges of pigs and onsite deliveries therefore potentially spreading disease. After the incubation period, disease symptoms trigger higher safety and biosecurity protocols such as interruption of animal movements, and therefore a decrease in the chance of disease spread.

#### Human behavior

Experimental gaming data collected by the Social-Ecological Gaming and Simulation (SEGS) Lab have shown that low compliance with biosecurity protocols by workers, poor biosecurity planning by managers or weak biosecurity messages from the leadership, create vulnerabilities in the production system leaving it more exposed to biosecurity breaches and diseases (8–12, 49–51, 53, 54). The experimental games also allowed us to identify behavioral trends within the game data, finding three distinct clusters which differ according to risk attitude (8). Risk attitude can be understood as an individual's positive (favorable) or negative (unfavorable) evaluation of taking risk when there is a disease outbreak. Risk-averse individuals tend to invest in biosecurity even when no or few infections are present in the network (risk of infection almost inexistent). “Opportunists” are willing to invest in biosecurity as risk arises meaning that they don't invest in biosecurity when perceived risk is low but begin to invest more heavily when risk is high. Finally, risk-tolerant are hardly willing to invest in biosecurity unless the epidemic environment is perceived as extremely dangerous. Consistent with these results, our ABM was built on the assumption that biosecurity compliance and investments can lead to improvements in both animal health and production. We developed three main mechanisms in the model that modulate how agents' risk attitudes and decision heuristics influence biosecurity: biosecurity investments, biosecurity compliance and risk attitude learning.

Producer agents can choose whether or not to invest in biosecurity based on disease status updates (number of infected premises in the network) sent by their veterinarian agent. Specifically, each agent's probability of investing in additional biosecurity is governed by a logistic function and depends on both the number of infected farms within its veterinarian network and the agent's risk attitude. These heuristics are based on data collected by SEGS showing that as infections on nearby farms increase, managers will become more likely to invest in biosecurity, even if it means taking a financial hit up front (8, 51).

Biosecurity compliance is modeled based on the phenomenon of psychological distancing whereby the longer in the past an event occurred, the less likely it is to be a salient decision factor (55–57). Experimental games showed that compliance with biosecurity protocols on the part of farm employees tends to fall off the longer it has been since the farm experienced an infection event (9, 49). In the ABM, we encoded this phenomenon with a linear decrease in biosecurity over time for as long as a producer is not symptomatic, and when a producer enters the symptomatic state the biosecurity decline is suspended.

The producer agents can change their initial risk-attitude over the course of a simulation in the current ABM, while in previous versions, hog producers had fixed risk attitudes. Risk attitude changes are encoded with an agnostic learning process meaning the risk-attitude shifts were derived statistically from experimental games and assigned to each ABM agent at model start. The learning is not generated contextually in the simulation as a response to interactions with the model environment. The decision to add this human behavioral mechanism as an agnostic learning reflects our awareness of the complexity of potential drivers behind risk-attitude changes. We know from literature and our experimental-game results that humans can change their response to risk, adapting it to what they experience and the information they receive (58, 59). Risk-attitude may change depending, for example, on combined effects of previous disease experiences, frequency of disease occurrence, work role, personal investment and responsibility with disease handling. For instance, a producer who has successfully dealt with disease in the past might learn that disease is not such a risk and become more risk tolerant in the future. Our experimental game emulated the biosecurity investment decision making process, allowing participants to react to various outbreak scenarios by choosing whether or not to invest in biosecurity to mitigate the probability of infection (8, 51). Using a large sample of online participants, we quantified distributions of behavioral risk with respect to information regarding disease incidence and biosecurity protection across a simulated supply chain. This also allowed us to assess how our participants transition to more or less risky behaviors throughout their simulated experiences. These distributions of observed risk attitudes and their behavioral trajectories were then embedded within the design of our model agents.

#### Individual model simulations

A simulation experiment is devised to examine the agents over time in order to explore the effect of animal and feed movement and individual risk attitudes on the disease spread. Upon initialization, each individual agent is assigned a role in the production chain (hog production premises, feed mill or slaughter plant) and in the case of hog producer premises agent, a unique risk attitude (averse, opportunist, or tolerant) and a risk-attitude learning rule of either remaining in the same risk group or moving to a different one. Model simulations are run on a daily step. A simulation starts with an initializing phase of 1,610 days (232 weeks) after which a disease outbreak is started and the simulation continues for a total of 2,982 days (196 weeks) producing data for analyses. Thus, simulation outputs match the number of weeks from the observed dataset (1 June, 2014 to 25 February, 2018), which was published by the United States Department of Agriculture (Swine Enteric Coronavirus Disease Situation report—March 2018, http://www.aphis.usda.gov/animal-health/secd). The output variables were measures of disease and biosecurity.

### Model analyses

Our analysis followed three steps: first the calibration the ABM, then a sensitivity analysis and finally experiments with risk-tolerance scenarios related to our research questions. The simulations for these model analyses were conducted in AnyLogic. Experimental scenarios for both the sensitivity analysis and risk-tolerance analysis consisted of Monte Carlo (MC) simulations with 500 replicates with different random seeds. MC experiments output weekly time series of three variables of interest, disease incidence, number of biosecurity investments and average biosecurity at the system level. The simulated data were collected and exported to files for statistical analysis and visualization.

We processed output data using R (60) and R Studio (61). We analyzed the variables' time series as well as summary variables such total incidence, total biosecurity investments and average biosecurity over the simulation time. The summary variables were calculated as the sum over a time series in the case of disease incidence and biosecurity investments and as the average across a time series in the case of average biosecurity. We compared variables' distributions across scenarios using non-parametric tests because the data did not meet either the assumption of normality (Shapiro-Wilk test) or equal variances (Brown-Forsythe test and Fligner-Killeen test). A pairwise comparison with the two-sample Kolmogorov-Smirnov test evaluated differences across scenarios for these output variables and box-plots were used to display outputs. For the risk-tolerance analysis, we also calculated linear regression models on the time series of incidence fitting separately each MC replicate for every scenario. We compared the distributions of regression slopes in the same way as we compared the other output variables to test whether the disease decreased (negative regression slope) or not in the system over the simulated time.

#### Calibration

We calibrated the ABM by matching the weekly incidence from the modeled system and the observed reality, so that the simulation can be closer to the actual case. The analysis was performed in the AnyLogic software environment using a built-in OptQuest genetic algorithm to minimize the Root Mean Square Error (RMSE) between the modeled and observed incidence over the historical period 05/31/2014 to 02/25/2018. By calibration, we determined four model parameters which control human behavior and about which we lacked data: the increase of biosecurity for each investment, the rate of biosecurity decline due to lack of compliance (psychological distancing), relative difference in the number of infected agents necessary to trigger a biosecurity investment for the three risk attitude groups, and the minimum time lapse allowed between biosecurity investments. We proceeded by a first calibration with larger ranges of search and then narrowed those ranges around the optimized parameter values for a more refined calibration. Each calibration scenario was run with 100 replicates.

#### Sensitivity analysis

With the sensitivity analysis, we wished to understand the model behavior in response to the variation of four model parameters directly related to disease incidence (Table 1). We used the calibrated model as reference and tested conditional variability in incidence by varying one parameter at a time keeping the others at baseline values. No interactions between parameters were tested.

**Table 1:** Variables used to test model sensitivity.

**Parameter**	**Tested parameter values**				
Biosecurity increase at investment time	0.01	***0.3***	0.6	0.9	
Biosecurity compliance—decrease rate (Δbiosecurity/week)	0	***−0.001***	**–**0.005	**–**0.01	
Disease subclinical incubation time (days)	0	***2***	7	14	21
Initial infections: Hog farm; feed mill; slaughter plant infections	*3; 1; 0*	15; 5; 0	30; 10; 0	60; 20; 0	

In italic bold are the values of the baseline model obtained from the calibration and used as reference for the analysis. Variables were varied one at a time keeping all the others at the baseline value. Each table cell represents therefore a sensitivity scenario run independently with 500 replicates.

#### Risk tolerance scenario experiments

The potential for widespread epidemics, delayed biosecurity protection and unpredictability of disease spread accompanying risk tolerance were explored *via* a series of simulation experiments. We were interested in examining a simulated producer population that reflected conditions of relatively high-risk tolerance in the system. The decision followed from conversations with veterinarians who witnessed the PED outbreak and became aware that producers were not ready with the appropriate biosecurity to face the unexpected and virulent disease. We set up three experiments to analyze incidence and biosecurity in simulated systems and we implemented three ABMs where we varied different model parameters and/or mechanisms that control risk behavior (Tables 2, 3):

1. Experiment 1: We compared the baseline system with an initial (naïve) hog producer agent population of 33% averse, 33% opportunist, 33% tolerant to a more risk tolerant system with an initial population of 0% averse, 50% risk opportunist, and 50% risk tolerant. All the simulations were run with the risk-attitude learning mechanism on. The ABM is expected to provide an estimate of the change in disease incidence related to different initial conditions.
2. Experiment 2: Initializing the model with a more tolerant population (scenario 2 of experiment 1), we varied the parameter x_0_, which represents the number of infected producer agents (infected hog facilities) needed to trigger an investment in biosecurity with a probability of 0.5. In a set of 11 scenarios, the parameter x_0_ is varied from 3.36 to 20.86 in increments of 1.75 for both tolerant and opportunist and is fixed at 0.14 for averse agents (Table 3). Despite its meaning, the x_0_ values are not integer numbers because they are rescaled for the model where they are set through an ancillary parameter linked to the probability function of biosecurity investment (see ODD + D). All the simulations were run with the risk-attitude learning mechanism on. This ABM is expected to estimate the effects of delaying biosecurity investments due to risk tolerance.
3. Experiment 3: Building on experiment 1 and 2, we compared the effects of the presence and the absence of the risk-attitude learning mechanism. We selected scenarios 1, 6, and 11 from experiment 2 (Table 3) with low, intermediate and high x_0_ values, respectively, and initialized the model with a producer agent population of 50% tolerant and 50% opportunistic. In each of the previous experiments, the ABM with the risk-attitude learning mechanism was set to lead to a final population with 47% risk averse, 30% opportunist, and 23% tolerant (Table 4). When the learning mechanism is off, the population keeps the initial risk attitude distribution for the entire simulation time. This ABM is expected to provide information on the effect of the opposing force provided by risk-attitude learning against the dynamics triggered by tolerant behaviors.

**Table 2:** Summary table of the three experiments run to test effects of increased risk tolerance on simulated disease spread in a hog production system.

**Scenario variables**	**Initial risk attitude**	**Risk tolerance: x_0_ center tolerant **	**Risk attitude **
**Experiment**	**proportions**	**and x_0_ center opportunist**	**learning**
1: Naïve vs. tolerant population	**Varied:** - Naïve: 33.3% averse, 33.3% opportunist, and 33.3% tolerant. - Tolerant: 0% averse, 50% opportunist, and 50% tolerant.	**Fixed**: x_0_ tolerant = 3.36 x_0_ opportunist = 1.75	**Fixed:** Learning mechanism ON
2: Risk tolerance increase (x_0_)	**Fixed**: Tolerant: 0% averse, 50% opportunist, and 50% tolerant.	**Varied**: See Table 3	**Fixed**: Learning mechanism ON
3: Risk-attitude learning vs. no-learning	**Fixed**: Tolerant: 0% averse, 50% opportunist, and 50% tolerant.	**Varied**: Three combined cases from Table 3:1. x_0_ tolerant = 3.36;2. x_0_ opportunist = 1.75 3. x_0_ tolerant = 12.11, x_0_ opportunist = 6.125 x_0_ tolerant = 20.86, x_0_ opportunist = 10.5	**Varied**: - Learning mechanism ON - Learning mechanism OFF

**Table 3:** Values of x_0_ used for the experiment scenarios.

**Scenario**	**x_0_ center tolerant**	**x_0_ center opportunist**	**Learning mechanism**	**Old scenario name**
Baseline	3.36	1.75	Yes	
1	3.36	1.75	Yes	NI048
1 NL	3.36	1.75	No	
2	5.11	2.625	Yes	NI073
3	6.86	3.5	Yes	NI098
4	8.61	4.375	Yes	NI0123
5	10.36	5.25	Yes	NI0148
6	12.11	6.125	Yes	NI0173
6 NL	12.11	6.125	No	
7	13.86	7	Yes	NI0198
8	15.61	7.875	Yes	NI0223
9	17.36	8.75	Yes	NI0248
10	19.11	9.625	Yes	NI0273
11	20.86	10.5	Yes	NI0298
11 NL	20.86	10.5	No	

These values increase in bins of 1.75 and correspond to the number of infected producers within a producer agent network that will trigger an investment in biosecurity with a probability of 0.5. All scenarios have a producer agent population composed by 0% averse, 50% opportunist and 50% tolerant except for the baseline scenario that has an evenly distributed population with 1/3 of producers in each risk group.

**Table 4:** Initial and final proportion of producers in each risk attitude group when the risk-attitude learning mechanism is turned on.

**Risk attitude group**	**Averse**	**Opportunist**	**Tolerant**
**Time phase**			
Initial population	0	50%	50%
Final population	47%	30%	23%

## Results

### Calibration

**Figure 3:**
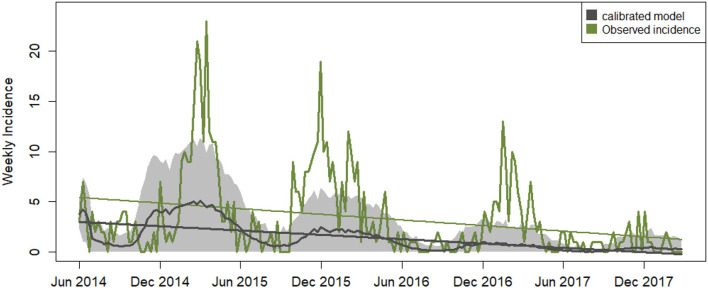
Weekly time series of disease incidence. Data related to the calibrated model outputs are shown in black: average modeled disease incidence (non-linear black line), its standard deviation generated across 100 iterations (gray shades) and the linear trend with an average slope of −0.017 ± 0.017. The green line depicts the observed incidence data for North Carolina with a linear trend superimposed of slope −0.021.

The calibration of the ABM optimized all four parameters with a minimum RMSE of 3.275 in incidence units (Table 5). This means that modeled incidence matched with ≤ 14% error the observed incidence, which ranges between 0 and 23 weekly new infection cases. The parameter combination was carefully cross-checked by running several calibration experiments with different starting parameter values and ranges. The search process yielded other possible parameter assignments with values close to the selected ones but higher RMSE. The calibrated model reproduces the seasonal variability and the negative trend of the observed data but on average, it misses the high winter peaks in incidence (Figure 3).

**Table 5:** Model calibration results: Optimized values for four parameter values linked to human behavior.

**Calibration parameter**	**Best parameter combination**
Biosecurity increase at investment time	0.3
Biosecurity investment—min. time interval (days)	28
Relative shift of investment probability across risk groups	0.07
Biosecurity compliance—decrease rate	−0.001
Objective value based on RMSE metric	3.275

The last row reports the root mean square error (RMSE) for the optimized parameter combination.

### Sensitivity analysis

**Figure 4:**
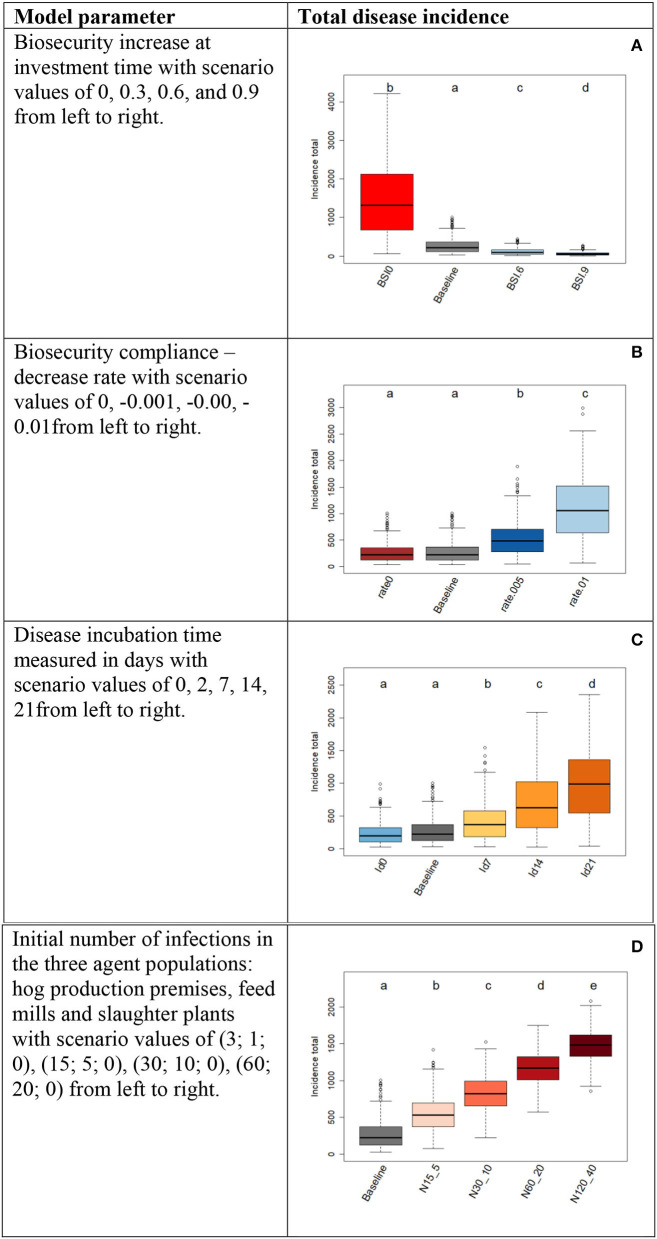
Results from sensitivity analysis. Each table row represents a sensitivity experiment where one model parameter was varied across a range of values (scenarios) as indicated in the left column. Capital letters distinguish the experiments: **(A)** for the parameter “Biosecurity increase at investment time”; **(B)** for “Biosecurity compliance”; **(C)** for “Disease subclinical incubation time” and; **(D)** for “Initial number of infections”. Box plots display the distributions of disease incidence totals with letters indicating significance from the pairwise distribution comparisons of scenarios. The baseline was used as reference scenario. Distributions not sharing any letter are different by the two-sample Kolmogorov-Smirnov test at the 5% level of significance.

Figure 4 displays the sensitivity analysis outputs and can be read as follows. The incidence distribution for the baseline model parameterization can be considered as reference and the other incidence distributions as outputs from different parameterization scenarios. We observed that changes in incidence are significant and follow the directions expected in the real-world observations. Only in two cases (Figures 4B, C)

a change from the baseline to lower parameter values leads to the correct direction of incidence change, but this change is not significant. In the case of biosecurity compliance (Figure 4B), this means that removing the mechanism of psychological distancing (compliance decrease rate = 0) will not significantly change incidence totals compared to those obtained from the baseline model with a compliance decrease rate = −0.001. However, more negative decreased rates of compliance do have significant increasing effects on incidence. In the case of disease subclinical incubation time, 2 vs. 0 days does not significantly influence the total incidence when the model is initialized with the baseline parameterization (Figure 4C). However, incubation times ≥ 7 days lead to significant increase in incidence. In summary, we confirmed that the disease mechanisms built in the model and controlled by these four parameters follow realistic behaviors, and we can trust the underlying model mechanisms controlled by these parameters.

### Risk tolerance scenario analysis

**Figure 5:**
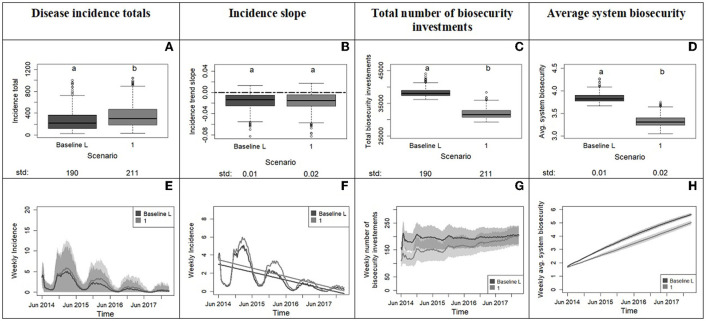
Outputs from experiment 1 comparing the system with the baseline population (33% averse, 33% opportunist, 33% tolerant) to a system with a more risk tolerant population (0% averse, 50% opportunist, 50% tolerant). The top row displays the box plots of system variables with the x axis showing the scenarios and the y axis reporting values for disease incidence totals **(A)**, incidence slope from time series **(B)**, total number of biosecurity investments **(C)** and average system biosecurity **(D)**. Letters on top of box plots report results from pairwise distribution comparisons of baseline against the tolerant scenario. Distribution not sharing any letter are different by the two-sample Kolmogorov-Smirnov test at the 5% level of significance. The standard deviation value of each distribution is printed below the box-plot x axis. The bottom row of the figure **(E–H)** displays the time series with weekly frequency for the variables; the colored shades show the variability provided by the Monte Carlo replicates. Panel **(F)** zooms into the average weekly incidence lines of panel **(E)** and includes the linear trends.

**Figure 6:**
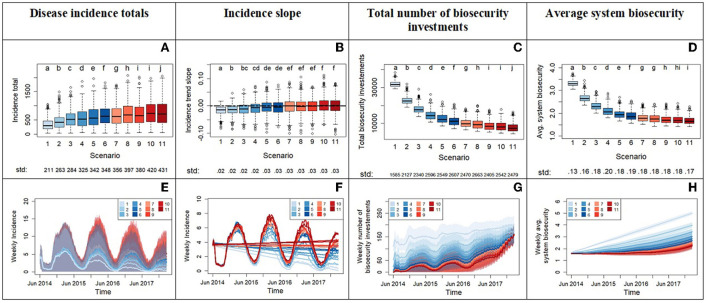
Outputs from experiment 2 comparing the system across a gradient of increasing tolerance with scenario 1 having the lowest tolerance and scenario 11 the highest. This is done by increasing the parameter x0, which represents the threshold in number of infected premises in the neighborhood of a producer before triggering the producer's investment in biosecurity. The top row displays the box plots of system variables (y axis): disease incidence totals **(A)**, incidence slope from time series **(B)**, total number of biosecurity investments **(C)**, and average system biosecurity **(D)**. The x axis reports the different scenarios. We applied a Bonferroni adjustment and test each hypothesis at level alpha = 0.0045. Letters on top of the box plots report results from pairwise distribution comparisons across scenarios. Distribution not sharing any letter are different by the two-sample Kolmogorov-Smirnov test at the alpha level of significance. The standard deviation value of each distribution is printed below the box-plot x axis. The bottom row of the figure **(E–H)** displays time series with weekly frequency for the variables. The colored shades show the variability provided by the Monte Carlo replicates. Panel **(F)** zooms into the average weekly incidence lines of panel **(E)** and includes the linear trends.

**Figure 7:**
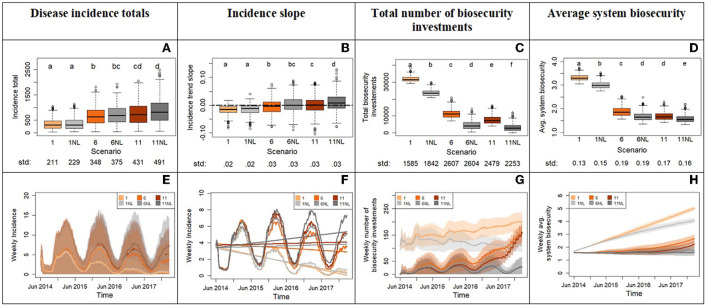
Outputs from experiment 3 comparing the system with and without risk-attitude learning mechanism for three different scenarios of experiment 2 (1, 6, and 11). The top row displays the box plots of system variables (y axis): disease incidence totals **(A)**, incidence slope from time series **(B)**, total number of biosecurity investments **(C)**, and average system biosecurity **(D)**. The x axis reports the different scenarios. We applied a Bonferroni adjustment and test each hypothesis at level alpha = 0.0083. Letters on top of the box plots report results from pairwise distribution comparisons across scenarios. Distribution not sharing any letter are different by the two-sample Kolmogorov-Smirnov test at the alpha level of significance. The standard deviation value of each distribution is printed below the box-plot x axis. The bottom row of the figure **(E–H)** displays the time series with weekly frequency for the variables. The colored shades show the variability provided by the Monte Carlo replicates. Panel **(F)** zooms into the average weekly incidence lines of panel **(E)** and includes the linear trends.

Using the ABM, three experiments were conducted, which systematically swept across treatments varying three parameters that control risk tolerance: the relative proportion of producer agents among the risk-attitude groups, the number of disease cases necessary for a producer to make a biosecurity investment and the ability of producers to change their risk attitude (risk-attitude learning). We summarize the results in the graphs displayed in Figures 5–**7**. The time series in these figures (panels E, G, and H) report the lines for the averages across the 500 Monte Carlo replicates and the shaded areas show the standard deviations. Our simulated data show that the system's epidemiological resilience can be significantly affected by changes in these parameters.

From Experiment 1, we learn that an initial absence of averse producers along with a higher number of tolerant and opportunist producers in scenario 1 cause a significant increase in the total incidence compared to the baseline scenario (Figures 5A, E). In the two scenarios, incidence decreases with the same slope, but this decrease takes longer in the more tolerant system. This outcome can be related to the lower average level of biosecurity in the more tolerant scenario due in part to fewer biosecurity investments (Figures 5C, D). Although the number of biosecurity investments toward the end of the simulation time in the two scenarios tends to converge, the biosecurity effect on disease is delayed in the tolerant scenario (Figures 5G, H). In summary, a change from a model initiated with a population of hog producers evenly distributed across the risk attitude groups (33% averse, 33% opportunist, and 33% tolerant) to a model initiated with a population of hog producers more tolerant and unprepared to a new disease (50% opportunist and 50% tolerant) means lowering the initial biosecurity in the system and that leads to longer times to boost biosecurity defenses and control infection.

Experiment 2 starts with the more tolerant risk attitude distribution from Experiment 1 and varies the trigger point x_0_ for biosecurity investment. Results (Figure 6) indicate that increasing tolerance to the number of infections needed to trigger biosecurity investments leads to two main outcomes: a significant increase in disease incidence and an overall increase in incidence variability. Higher variability means that the system becomes more unpredictable and susceptible to experience widespread epidemics with the introduction of a new disease (Figures 6A, E). This experiment reveals a switch in the epidemic patterns around scenarios 5 and 6, as demonstrated by the median regression slopes (Figures 6B, F) transitioning from negative to positive values:

- In scenarios numbers ≤ 6, the median of the incidence regression slope is < 0, meaning a reduction of disease incidence over the simulation time. We learn that values of the parameter x_0_ set to ≤ 12 neighbor infections for risk tolerant agents, and ≤ 6 infections for opportunist agents are necessary for negative slopes (median values). According to the medians, higher x_0_ values delay but do not prevent control of disease in this set of scenarios. These threshold values of x_0_ are not sufficient however to always assure disease control and cases of pandemics (positive slopes) can still happen as shown by the variability across Monte Carlo replicates (Figures 6A, B, E).
- In scenarios numbers > 6, the median of the regression slope is ≥0, meaning no decrease of incidence over the simulation time. Positive slopes indicate that the disease incidence cannot be controlled and may increase over the simulation time. Slopes close to zero are still an indication that the disease does not subside, remaining at initial levels, with seasonal variations, despite investments in biosecurity made by agents. In these scenarios, the higher values of the parameter x_0_ cause hog producers to react relatively slowly to disease presence and significantly restrain the chance to control an outbreak. The graphs highlight a rushed increase in biosecurity investments toward the end of the simulation time (Figure 6G), but it is unsuccessful at raising biosecurity to levels that contain disease (Figures 6A, H). The counteraction of risk-attitude learning in rising risk aversion in the system is not strong enough to prevent large percolations of disease as proved by experiment 3.

Experiment 3 shows that removing risk-attitude learning does not significantly change the total incidence for any scenario pair despite significant differences in total biosecurity investments and average biosecurity across scenarios (Figures 7A, C, D). It also does not significantly affect slopes except for the paired scenarios 11 and 11NL where learning produces flat median incidence trends while its absence positive ones (Figures 7B, F). This means that the risk attitude as parameterized in the ABM is generally not able to reverse trends set by initial conditions (Figures 7A, B, E). A notable outcome of this experiment is that the total incidence, slope and average system biosecurity for scenario 6NL and 11 are not significantly different despite higher investments in biosecurity in scenario 11. This means that learning risk aversion in a system with high risk tolerance to disease vicinity (scenario 11) reduces the impact of disease to the level of a system that does not learn risk aversion but starts with an intrinsic lower risk tolerance for disease (lower x_0_, scenario 6NL). However, the same is not true between scenarios 6 and 1NL: the low values of x_0_ in scenario 1 protects the system from disease thanks to relatively quick investments in biosecurity and this is significantly more effective than learning, which takes time and therefore delays its benefits for advancing biosecurity (Figures 7B, F). These results highlight the strong non-linearity of this modeled system.

## Discussion

The PEDv epidemic that began in 2013 provides both a historic example of multi-year disease exposure and an opportunity to learn from experience as food animal industries prepare for potential new FAD in the future. FADs are a concern because they can lead to instability in people's jobs, losses in income and an unsustainable use of economic resources from both production stakeholders and governmental entities in charge of stabilizing socio-economic functions. Recognition of FADs' negative and far-reaching consequences has prompted our research and modeling work on biosecurity and human behavior.

Koliba et al. (24) discuss how micro-level behaviors scale-up to larger macro-level patterns influencing disease spread across operational, tactical and strategic levels. Our agent-based model (ABM) integrates insights from the operational and tactical levels of analysis to inform a systems-level perspective accounting for decision making on biosecurity. The operational level is the farm employee level, which largely concerns employees' decisions to put prescribed biosecurity protocols into action. The tactical level involves managers of a farm or a farm company with its production network and focuses on biosecurity investments and implementation of biosecurity measures. Finally, the strategic level involves decision makers who have power to influence long-term policies at macro-scale and are concerned with the complex interaction patterns between agents across the production chain. The three levels are interconnected and influence one another's decisions.

We built our ABM to explore interactions between epidemiological and human behavioral mechanisms. As a descriptive and demonstrative model, we used it to illustrate that patterns of interest can be produced through agent-level rules and interactions. In particular, the human behavioral component controlling decisions on biosecurity is shown to have a critical role in determining the outcomes of a disease outbreak, which otherwise would remain subject only to epidemiological rules related to seasonal infection variability. We found biosecurity interventions activated by risk aversion at the beginning of a simulated outbreak crucial to prevent long-term presence of disease or widespread epidemics in the system. Divergently, slow shifts toward more risk averse positions are mostly lagging to control disease and reduce the intrinsic unpredictability of a system left with low biosecurity protection.

The findings from the model experiments help answer our three research questions and demonstrate that the biosecurity decisions at the premises level have ramifications for the protection of the whole production system:

- Question 1: If we start the model with a risk tolerant rather than a risk naïve population of producers, how does disease incidence evolve after a breakout? The implications of experiment 1 are that the initial risk-attitude distribution in the system affects the evolution of the disease. In our case, the removal of risk averse producers (compared to 33% averse in the naïve population) reflects the assumption of a producer population rather unprepared and unaware of the risk of a new disease. The consequence was higher incidence but no major pandemics since the population raised biosecurity quickly enough. This requisite on biosecurity-response timing is crucial to disease control as shown by experiment 2 directed by our second question.
- Question 2: If we model biosecurity investment at increasingly higher levels of tolerance (delayed investments), do we observe a tolerance scenario where percolations of disease in the system (pandemics) become the majority of simulation outcomes? In the scenario comparisons (Figure 6), we identified a range of x_0_ values (10–12 neighboring infections for risk tolerant and 5–6 for opportunist) that mark a transition from a system capable of controlling disease to one more prone to major disease percolations. In real-world situations, these specific x_0_ values might not apply, but this finding warns us that the delay of biosecurity implementation can hit a threshold beyond which disease control becomes severely harder and pandemics more likely. The model results also point out an increment in incidence variability associated with increased tolerance. The implications are that the disease dynamics in system become more unpredictable and therefore more difficult to control with biosecurity. We want to further reflect on this last point by focusing the attention to the rushed increase in biosecurity investments toward the end of the simulation time (Figure 6G), which was unsuccessful at reaching biosecurity levels adequate for containing disease and preventing pandemics (see median trends in Figures 6E, F, H). We can compare this investment behavior to a hasty emergency response adopted at a point where disease has become so prevalent that many producers become aware of risk of infection or are imposed mandatory biosecurity protocols by policies. In either case, urgent and hasty investments do not guarantee immediate disease control.
- Question 3: How important is learning risk aversion to counteracting initial conditions of risk tolerance? The data provided an unexpected weak effect of the learning mechanism on disease dynamics. For delivery of an effective and immediate response to a disease outbreak, the learning mechanism is not the one to count on. As modeled in the ABM, risk-attitude learning is a relatively slow process that shows its results over the course of years. We can think of learning as a mechanism that modifies risk culture and needs more time than mechanisms imposing quick behavioral changes such as policies. It is however a critical mechanism necessary in the real world because a cultural transition based on learned behaviors will prepare producers to prompt risk averse and biosecure responses.

From the ABM simulations emerged phenomena that corroborated the importance of human decisions and demonstrated complex interactions between epidemiological and human behavioral mechanisms. The present work highlighted complex behaviors driven by path-dependencies and non-linear dynamics. Path-dependency happens when present events and decisions condition the path of later events or decisions (62). In other words, the evolution of a path-dependent system is bound to its own history. In Experiment 1 for example, the two different initial distributions of risk attitudes determine initial decisions on biosecurity investments (Figure 5G), which govern the system biosecurity and disease infections, ultimately leading to significantly different paths in the disease evolution and outcome. Experiment 2 demonstrated a non-linear response of disease to a linear increase of risk tolerance. An increase in disease tolerance within relatively less risk tolerant populations triggered a higher biosecurity response, a higher increase in disease incidence and greater loss in disease control capacity (Figure 6, scenarios 1–6) than the same increase in disease tolerance applied in a population initially characterized by higher tolerance (Figure 6, scenarios 6–11). Figure 6 shows this non-linear saturation effect that reflects an increasingly reduced capacity of disease to spread in the system and trigger more biosecurity investments. The system approaches its biosecurity investment capacity. This close-to-saturation state does not mean that almost all agents are infected but rather that key nodes of disease transfer in the networks are infected and have already made the biosecurity investments allowed by the model rules (48).

Compared to previous ABM versions that advanced studies on network dynamics and effects of risk-attitude configurations (47, 48, 63), the current ABM, geared with the risk-attitude learning mechanism, allowed us to explore deeper and more complex questions on how diverse, connected and interdependent local actions have global impacts. Learning takes place over the duration of the 4-year simulation time in a step-wise fashion and as such, it can be interpreted as a process leading to a new risk-attitude culture in the system. Our results indicated that slowly learning risk aversion is not effective at counteracting risk tolerant behaviors that are set in a system where disease is already spreading. However, we believe that this cultural aspect of biosecurity is important because of its power to change risk culture over the long term. A biosecure culture, meaning a collective endorsement of preventive measures, can contribute to a more expansive sense of what is possible in the event of a disease incursion and therefore what could be invested now to be ready in the future. In this context, we recognize that culture and risk attitude are descriptive labels for complex mechanisms underlying people's behaviors. Therefore, the risk attitude behaviors and learning that we encoded in our ABM likely encompass a wider spectrum of human decision-making drivers. For example, hog producers who hardly respond to disease vicinity with biosecurity might behave so due to inability to access biosecurity resources or because of absence of economic means to make biosecurity investments.

Following points raised by An (64), we want to reflect on limitations of our model. While developing more holistic versions of the ABM by including human behavior, we have been incorporating rules based on data from experimental games but also hypothetical rules based on expert opinion in places where adequate data did not exist. For example, agents are infected in a stochastic manner with probabilities that depend on the type of contacts made between agents and were estimated based on expert opinion. Calibration-based estimates have been used to parameterize the model. For example, three parameters of the probability function controlling the decision on biosecurity investments were estimated *via* model calibration. There are clearly limitations and disadvantages associated with parameterizing the model with assumptions and/or calibration-based values as they may not be appropriate. Agent-based models deal with the study of socio-ecological systems that can be conceptualized through a set of micro and macro relationships and rules that leads to a complex internal ABM architecture. In a calibration setting, it is challenging to estimate the microscopic (agent-related) parameters directly from the macroscopic level (system incidence) because the large number of model parameters can result in a “curse of dimensionality” that lead to a multiplicity of possible parameter combinations. We minimized this issue by limiting the number of calibration-based parameters and used expert opinion to estimate a subset of parameters. We are therefore aware that parameterization of behavioral rules in the ABM that we set with these approaches should be used with caution.

The structure of the animal, feed and veterinarian networks was built on nearest neighbor metrics. For example, a farrow-to-wean producer agent will move its weaned pigs within a nearest-neighbor network of wean-to-feeder or wean-to-finish producers in the model but in the real world, a production company might have its premises distributed over large geographic areas and beyond neighboring premises. The modeled network assumption may lend to system emergent properties that are not fully representative of actual dynamics, which could include disease spread over larger distances. As a potential consequence, the disease transmission in our results is more localized and does not account for infections brought in and out of state. Nonetheless, we trusted model patterns based on the rationale that model calibration provided a parameterization that realized incidence values and a trend comparable with observations and the model validation confirmed realistic patterns.

In summary, our work corroborated the view that epidemiology and psychology need each other and we highlight three main points learnt from these modeling experiments:

- The initial biosecurity settings in the system play a key role in determining the evolution of disease spread. An initially risk tolerant population with low biosecurity preparedness will lead to prolonged time needed for disease control.
- Delayed biosecurity interventions due to disease tolerance leave the system vulnerable to unpredictable disease spread and higher likelihood of widespread epidemics.
- A slow change toward more averse behaviors (cultural change) does not significantly help disease control once the disease has spread in the system. However, efforts directed at creating a risk averse culture are essential to prepare hog producers (and other stakeholders) with the level of biosecurity necessary to promptly counteract the incursion of a potential new disease.

## Conclusions and future research

Controlling the emergence, spread, and persistence of infectious animal diseases is a significant challenge in our interconnected world. Managing animal diseases requires stakeholders of the production chain as well as policy makers to adopt a perspective that adequately represents the complex causal relationships between disease transmission and human behavior. Agent-based modeling is a powerful method to study complex systems such as the animal production chain and simulate epidemiological and human interactions. This paper explored questions on the coupled epidemiological-human behavioral aspects that influence disease control. The results show that a strong initial response with biosecurity investments associated with low risk tolerance is more effective than a steady shift toward a more risk averse culture in the face of a new infectious disease such as PEDv. A clear danger for a system with low biosecurity levels is that disease dynamics become highly variable and unpredictable. This means that delayed biosecurity interventions, even if rapidly implemented, might not prevent occurrence of widespread epidemics.

Our ABM is still under active development, and as such there are a number of features we intend to implement. We are working to link agents' biosecurity decision-making to a budgetary/economic sub-model, since, in the real world, a hog producer could not implement a new biosecurity protocol without sufficient cash or capital on hand for a bank loan. We also will scale up the model to include system dynamics across the entire United States. It is vital that both the scientific community and the industry embrace and adopt innovative and advanced research methods to identify the best directions for research, practice, and policy to support the resilience of food animal production in the face of emerging and exotic disease threats. Disease transmission takes place within social-ecological systems and therefore, aspects of human behavior are an essential construct in modeling work for understanding disease dynamics. Their incorporation in our agent-based model lead to robust insights on how personality and variability in behavior can lead to very different outbreak signatures that require attentive interventions and preparedness plans.

## Data availability statement

The raw data supporting the conclusions of this article will be made available by the authors, without undue reservation.

## Author contributions

GB, EC, SM, and JS contributed to conceptualization and writing of original draft. GB, SM, EC, SW, AZ, CK, and JS contributed to conceptualization, investigation, methodology, project administration, supervision, and writing—review and editing. GB, SM, EC, SW, and LT contributed to conceptualization, software, and writing—review and editing. GB, SM, OL-C, and NC contributed to software parameterization and review. JS, AZ, and CK contributed to investigation, funding acquisition, project administration, and writing—review and editing. All authors contributed to the article and approved the submitted version.
