# Supplementary material for: Connecting livestock disease dynamics to human learning and biosecurity decisions
Source: Front Vet Sci. 2023 Jan 20;9:1067364. doi: 10.3389/fvets.2022.1067364 (PMC9896627; doi:10.3389/fvets.2022.1067364)
Supplement: Supplementary file 1 [file Presentation_1.pdf]

ODD+D Protocol: Regional U.S. Hog Production Chain Biosecurity Model v.2.0.47

Gabriela Bucini, Plant and Soil Science Department, University of Vermont

December, 2022

This material is based upon work that is supported by the National Institute of Food and Agriculture, U.S. Department of Agriculture, under award number 2015-69004-23273. Any opinions, findings, conclusions, or recommendations expressed in this publication are those of the author(s) and do not necessarily reflect the view of the U.S. Department of Agriculture. The funders had no role in study design, data collection and analysis, decision to publish, or preparation of the manuscript.

The following model description follows the ODD (Overview, Design concepts, Details) protocol for describing individual- and agent-based models (*Grimm et al. 2006, 2010*), with ODD+D amendments as proposed by Müller et al. (2013).

I) Overview

I.i Purpose

I.i.a What is the purpose of the study?

The Regional U.S. Hog Production Chain Biosecurity Model (RUSHPCBM), version 2.0.40, is an agent-based model developed to assess both supply chain network level and human-behavioral factors relevant to the spread of socioeconomically-important diseases through the U.S. hog production chain. RUSHPCBM has been developed using AnyLogic v.8 software, which is in turn based on the Java programming language. The model is calibrated to represent hog production within the U.S. states of North Carolina, Iowa, and Illinois. These three states serve as case studies, since they are all major hog producers, while also having interesting supply chain network features that differentiate them from one another.

The purpose of the study is to advance understanding of human behavioral processes for prevention of disease in an animal production system. The ABM is instrumental in exploring patterns of disease spread emerging from many individual decisions and interactions among agents and their environment. Current disease models contain epidemiological processes but rarely consider simulating dynamics in risk perception and its impact on the adaptive biosecurity behavior. This ABM simulates decision-making strategies on biosecurity investment and compliance for agents based on several factors including risk attitude, learning of risk behavior and distribution of information about disease presence. The model aims to qualitatively reproduce and explain patterns observed in the outbreak of porcine epidemic diarrhea virus during 2014-2018 in the US. A hog production system is modelled to study the pathways of disease spread among agents in relation to their capabilities to adapt by changing their biosecurity level. Our ABM is essentially a combination of four functional components: production chain, epidemiology and human behavior related to biosecurity. Overlaid on the network structure of a simulated supply chain, the epidemiological component coupled with the human behavioral component

simulate both direct and indirect processes of disease transmission. The specific questions addressed are:

- What essential decision-making features need to be coded to simulate a realistic human adaptive behavior?
- What patterns emerge from adding the relationships linking biosecurity to risk attitude?
- How do agent adaptability and learning affect patterns of disease spread and incidence?

Model calibration was undertaken using available datasets coupled with an iterative expert informant advisory process. The model uses agricultural statistics and model calibration tools to generate realistic production chain networks of producers, feed mills, and slaughter plants within the spatial bounds of each study area state.
The epidemiological spread model is of the Susceptible / Infective subclinical / Infective symptomatic / Susceptible type, with infections transmitted between agents probabilistically based on patterns of trade and contact gleaned from industry experts and a review of the primary literature. Disease spread probabilities associated with the different types of inter-agent contact have been calibrated by reference to epidemiological data concerning disease spread dynamics associated with previous real-world epidemic events in the hog industry, as well as input from livestock veterinary professionals.

I.i.b For whom is the model designed?

The model is designed for use by university researchers, industry practitioners, veterinary specialists, agricultural extension practitioners and government agencies wishing to analyze the dynamics and consequences of disease spread in the U.S. hog production chain under varying assumptions concerning disease characteristics, production chain network structures and human behavioral implementation of biosecurity measures and agent behaviors that may prevent or curb catastrophic outbreaks.

<u>I.ii Entities, State Variables, and Scales</u>

I.ii.a What kinds of entities are in the model?

Four classifications of hog production chain network agents, identified by industry experts as critical players in the transmission of disease, are represented in the model. These are (a) producers, (b) feed mills, (c) slaughter plants, and (d) veterinarians. Producer agents are assigned one of six industry roles. Five of these encompass the USDA's classification system for hog producers, these being (a) Farrow to Wean, (b) Wean to Feeder (a.k.a. Nursery), (c) Feeder to Finish (a.k.a. Finish Only), (d) Farrow to Feeder, and (e) Farrow to Finish. Upon the advice of industry experts, an additional producer classification, (f) Wean to Finish — which has recently become more popular in the industry — is also included in the model. Figure 1 below shows producer, feed mill and slaughter plants agent types, their graphical representation in the model, and an outline of the heuristics that govern inter-agent contact patterns. Each veterinarian agent is connected with a number of producer agents through a service network. Veterinarians do not have a graphical representation in our model.

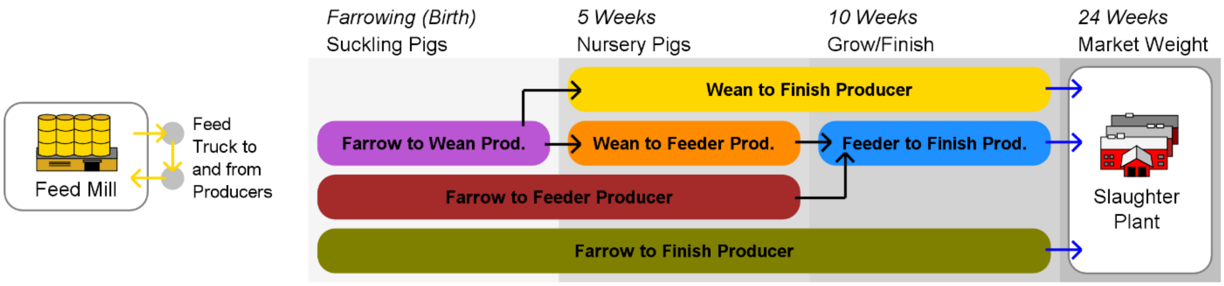

Figure 1: Structure of connections between agents, including hoofstock age transfer conditions where applicable.

I.ii.b By what attributes (i.e. state variables and parameters) are these entities characterized?

Each agent class has a specific set of state variables and parameters relevant to its industry role. These are given in Table 1 to Table 9, below.

Table 1**:** Parameters and variables common to all producer, feed mill and slaughter plants agents

Attribute	Description
Static parameters (set at initialization)	
My name	String representation encoding agent class and index (for tracking network connections)
State variables (may change throughout simulation run)	
Infectivity state	Either “clean” or “infected”.
Has been infected	Flag indicating whether the agent was ever infected during a run.
Total infection duration	Counter indicating the total number of days an agent was infected during a run.
Contact network out degree & in degree	List of the other agents with whom each agent had contact throughout a run, as well as the number of times contact between the two agents occurred. Out degree is incremented whenever an agent sends animals or feed; in-degree whenever animals or feed are received.
Infection network out degree	List of the other agents to whom an agent spread the disease, along with the number of times infection spread occurred between the two agents throughout a run.
Biosecurity level	Type double in range [0 – 8] indicating the agent’s biosecurity level, with 0 corresponding to the lowest level of biosecurity and 8 corresponding to the highest level of biosecurity. The biosecurity level is connected to the probability of getting/spreading infection, with the lowest

	probability at the highest biosecurity level (8) and viceversa.

Table 2**:** Parameters and variables for producer agents

Attribute	Description
Static parameters (set at initialization)	
Farm category	Encodes which of the 6 producer industry roles this agent falls into.
Total capacity	Total animal capacity.
Number of sows	Number of sows (calculated using parameters for the ratio of sows to pigs associated with each producer classification).
Non-sow capacity	Total capacity minus number of sows.
My transferee producers	List of potential producer trading partner agent objects.
My slaughter plant	Link to slaughter plant agent object (finishing producers only).
My feed mill	Link to feed mill agent object.
Initial Risk attitude	Risk attitude is a class variable. Each agent is initially assigned to one of three risk attitude groups including: risk tolerant, risk opportunist and risk averse.
Final Risk attitude	Risk attitude is a class variable. Through learning, agents can either change their risk attitude. Based on experimental game data, some agents change their risk attitude and by the end of the run and some remain with their initial risk attitude. The final risk attitude is one of three risk attitudes: risk tolerant, risk opportunist or risk averse.
Risk learning behavior	Risk learning behavior is a class variable. Each agent belongs to one of nine "risk learning behavior" groups. These groups are generated from the paired combinations of the three risk attitudes (risk tolerant, risk opportunist and risk averse) and describe a transition from a risk group to another through learning. For example "AverseToTolerant" is a learning group.
State variables (may change throughout simulation run)	
Current piglet inventory	Number of piglets currently available to wean and batch.
Last weaning day	Timecode of the last date piglets were batched and encoded in the pig batch tracker.
Current non-sow pig inventory	Number of pigs (that is, neither piglets nor sows) currently on premises.

Pig batch tracker	Non-sow pigs are represented by a data structure encoding the size of each batch of stock currently making up the agent's hoofstock inventory, along with a timestamp representing that pig batch's "birthday", which is used to calculate the age of the batch.
Pig shipments in & out	Lists of sizes (number of animals) of each shipment incoming from and outgoing to the producer (used for calibration).
Feed deliveries in	Number of feed deliveries incoming to the producer (used for calibration).
Potential biosecurity level	Level of biosecurity that a hog production premise can potentially achieve with full compliance. It ranges from 0 to 8 with 0 being no biosecurity implemented on the farm and 8 a very high level of biosecurity implemented on the farm.
Biosecurity level	Level of biosecurity of a hog production premise. It ranges range from 0 to 8 with 0 being no biosecurity implemented on the farm and 8 a very high level of biosecurity implemented on the farm. It can be lower than the potential biosecurity level if there is no full compliance (biosecurity discounting rate > 0).
Biosecurity Discounting Rate	Rate at which psychological distancing decreases biosecurity compliance. It depends on the infection risk level and it affects the actual biosecurity level of producer agents (not the potential biosecurity level).
Infection spread probabilities	Set by applying the infection probability functions that map the agent's biosecurity to infection probability. They vary as the agent's biosecurity changes through the run.

Table 3: Parameters and variables for feed mill agents

**Attribute**	**Description**
Static parameters (set at initialization)	
Infection spread probabilities	Set at initialization by applying the infection probability functions that map the agent's biosecurity to infection probability.
Biosecurity level	Level of biosecurity set at initialization. It ranges range from 0 to 8 with 0 being no biosecurity implemented in the feed mill and 8 a very high level of biosecurity.
Risk attitude	Risk attitude is a class variable and it is set at initialization. Each feed mill agent belongs to one of three risk attitude groups including: risk tolerant, risk opportunist and risk averse.
State variables (may change throughout simulation run)	

Truck infected	Flag indicating whether the feed mill's delivery truck is currently infected.
Feed deliveries out	Number of outgoing feed deliveries to producers (used for calibration).

Table 4: Parameters and variables for slaughter plant agents

Attribute	Description
Static parameters (set at initialization)	
Infection spread probabilities	Set at initialization by applying the infection probability functions that map the agent's biosecurity to infection probability.
Biosecurity level	Level of biosecurity of slaughter plant set at initialization. It ranges range from 0 to 8 with 0 being no biosecurity implemented and 8 a very high level of biosecurity implemented.
Risk attitude	Risk attitude is a class variable and it is set at initialization. Each slaughter plant agent belongs to one of three risk attitude groups: risk tolerant, risk opportunist and risk averse.
State variables (may change throughout simulation run)	
Pig shipments in	List of sizes (number of animals) of each shipment incoming from producers (used for calibration).

Table 5: Parameters and variables for veterinarian agents

Attribute	Description
Static parameters (set at initialization)	
Size of the service network	Set at initialization, the number of producer agents in the veterinarian service network.
Message delivery frequency	Time frequency at which the veterinarian agent send messages through its producer network with updates on infection status (number of infected production premises)
State variables (may change throughout simulation run)	
Messaging status	The veterinarian can be either idle or sending messages with infection data

I.ii.c What are the exogenous factors/drivers of the model?

Exogenous factors include:

- The operational distributions of agents of each class within each study area (Table 6 and Table 7Table 6)
- Disease related factors: general disease parameters, disease spread probabilities specific to each modality of inter-agent contact, disease seasonality, and parameters pertaining to each agent class (Table 8, Table 8).
- Access to disease information sent by veterinarian affecting decisions on biosecurity investment by producer agents.

Note: In the tables, "EAP" in data source column indicates that the value was derived through expert advisory panel sessions. "FHPC" refers to the family-owned hog production chain system dataset.

Table 6*:* Parameters remaining fixed throughout each model run

	Baseline Value(s)			
**Parameter**	***North Carolina***	***Iowa***	***Illinois***	**Data Source(s)**
Num. producers	2217	6266	2045	Burdett et al. (2015)
Avg. producer capacity	4015	3265	2264	Burdett et al. (2015)
Proportion farrow to wean	0.050	0.026	0.038	Burdett et al. (2015)
Proportion farrow to feeder	0.005	0.010	0.009	Burdett et al. (2015)
Proportion farrow to finish	0.554	0.304	0.635	Burdett et al. (2015)
Proportion wean to feeder	0.102	0.064	0.023	Burdett et al. (2015)
Proportion wean to finish	0.003	0.077	0.055	Burdett et al. (2015)
Proportion feeder to finish	0.286	0.519	0.241	Burdett et al. (2015)
Num. slaughter plants	24	18	25	USDA NASS (2014)
Num. feed mills	40	11	37	Google search; EAP

Table 7**:** Parameters common to all study areas, remaining fixed throughout each model run

Parameter	Description / Units	Baseline Value(s)	Data Source(s)
Disease parameters			
Percent to infect	Percent of producers initially infected	5%	-
Avg. producer infection length	Avg. duration of producer infection (days)	40	Goede & Morrison (2016); EAP
Avg. slaughter plant infection length	Avg. duration of slaughter plant infection (days)	5	EAP
Avg. feed mill infection length	Avg. duration of feed mill infection (days)	20	EAP

Suckling mortality rate	Proportion of suckling pigs dying if infected	.98	Goede & Morrison (2016); EAP
Nursery mortality rate	Proportion of nursery pigs dying if infected	.75	Goede & Morrison (2016); EAP
Grow/finish mortality rate	Proportion of grow/finish hogs dying if infected	.25	Goede & Morrison (2016); EAP
Producer farrow, wean, and batch parameters			
Farrow to wean sow proportion	Relative to total capacity	.6	EAP
Farrow to feeder sow proportion	Relative to total capacity	.5	EAP
Farrow to finish sow proportion	Relative to total capacity	.2	EAP
Annual piglets per sow	Number of piglets	34	The Pig Site (2014)
Max. frequency of weaning	"farrow, wean and batch" function freq. (days)	7	EAP
Min. batch size	As proportion of total capacity	0.05	EAP
Capacity under which one batch	Small producers have only one pig batch	20	EAP
Producer to producer transfer parameters			
Min. capacity similarity ratio	Trading producers cannot be greatly different sizes	25	EAP
Max. producer connection distance	Max. distance between trading producers (km)	150	EAP
Max. potential transferees	Max. number of producer trading partners	15	FHPC
Max. shipment frequency	"evaluate pig shipments" function freq. (days)	5	FHPC
Feed mill parameters			
Avg. daily trips	Avg. num. daily feed deliveries per mill	10	FHPC
Num. producers visited λ	Expected to visit λ per delivery (Poisson)	1	EAP

Table 8: State variables common to all study areas that may change throughout simulation run. Infection probabilities may change due to seasonal variability of virus infectivity and producers' biosecurity level.

**Parameter**	**Description / Units**	**Value at p_{max} (see Table 10)**	**Data Source(s)**
Disease state variables			
Producer disease spread probabilities			
Prob. producer will become infected if returning pig truck is contaminated		0.35	EAP
Prob. producer will become infected if delivered feed is contaminated		0.8	EAP
Prob. feed truck will become contaminated if producer is infected		0.15	EAP
Prob. pig truck will become contaminated if producer is infected		0.4	EAP
Prob. symptomatic pigs will infect transferee farm		1	EAP
Feed mill disease spread probabilities			
Prob. feed mill will become infected if returning feed truck is contaminated		0.25	EAP

Prob. feed truck will become contaminated if feed mill is infected	0.99	EAP
Slaughter plant disease spread probabilities		
Prob. slaughter plant receiving area will become infected if pig batch is infected	0.99	EAP
Prob. pig truck will become contaminated if receiving area is infected	0.25	EAP

Table 9: Parameters related to infection in the ABM. Parameters remain fixed throughout each model run

Parameter	Value
AverageLengthOfFarmInfectionDays	50
AverageLengthOfSlaughterPlantInfectionDays	60
SucklingMortalityRate	0.98
NurseryMortalityRate	0.75
GrowFinishMortalityRate	0.25
InitialHogFarmInfections	3
InitialFeedMillInfections	1
InitialSlaughterPlantInfections	0
InitialInfectionYear	2,014
InitialInfectionMonth	5
seasonalityOn	TRUE
peakMonth_seasonalityAdj	1
alpha_seasonalityAdj	0.5
min_Logistic	0.05
x0_Logistic	4
K_Logistic_FarmWillBeInfectedIfFeedDeliveryTrailerIsInfected	0.8
m_Logistic	1.3
K_Logistic_FeedDeliveryTrailerWillBeInfectedIfFarmIsInfected	0.15
K_Logistic_PigDeliveryTrailerWillBeInfectedIfFarmIsInfected	0.4
K_Logistic_InfectedPigsWillInfectSlaughterPlantReceivingArea	0.99
K_Logistic_InfectedReceivingAreaWillInfectPigDeliveryTrailer	0.25
K_Logistic_InfectedFeedDeliveryTrailerWillInfectFeedMillUponReturn	0.25
K_Logistic_FeedDeliveryTrailerWillBeInfectedIfFeedMillIsInfected	0.99
K_Logistic_PigGroupsWillBeInfectedIfAuctionHouseIsInfected	0.8
K_Logistic_PigDeliveryTrailerWillBeInfectedIfAuctionHouseIsInfected	0.25
K_Logistic_AuctionHouseWillBeInfectedIfFeedDeliveryTrailerIsInfected	0.25
K_Logistic_FeedDeliveryTrailerWillBeInfectedIfAuctionHouseIsInfected	0.25
K_Logistic_FarmWillBeInfectedIfReturningPigDeliveryTrailerIsInfected	0.35
percentFarmsWithEnvironmentalInfection	0.3
K_Logistic_RedenderingTruckWillInfectFarm	0.01
K_Logistic_VisitorWillInfectFarm	0.008

InitialInfectionDay	1,611
min_seasonalityAdj	0.3

I.ii.d If applicable, how is space included in the model?

The model is spatially situated in a continuous, two-dimensional GIS environment. Distances between agents are calculated “as the crow flies” and measured in kilometers. In some cases, distance is a factor in determining inter-agent contact patterns.

I.ii.e What are the temporal and spatial resolutions and extents of the model?

The model’s time scale is based on real-world days, with the initial model date set to December 27th, 2009. The year 2009 was selected to allow sufficient model time to stabilize the processes simulating the swine production chain before the disease outbreak event set in May 2014. The outbreak date matches the start of available disease incidence data. The model’s stop date can be set as desired depending on the experimental phenomena the user is interested in studying, with a default setting of 25 February, 2018 (last date of available disease incidence records). Model time is continuous (i.e., events may occur part-way through a day). The model’s spatial extents correspond to the extents of the selected U.S. state study area. Space is also continuous in the model, utilizing a two-dimensional GIS framework.

<u>I.iii Process Overview and Scheduling</u>

I.iii.a What entity does what, and in what order?

Five classes of functions define the operation of the model, presented in order of the point(s) in the simulation that they occur (see the *Implementation Details* section). First are the initialization functions, which define how the agents will be physically situated in the space, set each agent’s individual operational, behavioral and economic parameters, and identify lists of potential trading partners based on the classification and industry role of the agent, as well as spatial proximity to other agents. Second are the cyclically-executing functions, which make up the agents’ decision rules determining how and when contact between agents will occur (through the transfer of livestock and the distribution of feed), and thereby opening potentials for infection to spread. These functions also determine and implement the consequences of an infection upon the agent. The risk-attitude learning is also a cyclic function that modifies the probability of investment between according to the risk-attitude transition set for the producer agent at the initialization stage. Third is the initial infection function, which is called after the initial transient period in each run. The infection event triggers the veterinarian agent’s functions that collect and send disease information to producer agents. The delivered disease information along with the producer agent’s infectivity state trigger the human behavioral functions that determine the probability of biosecurity investments and the onset of psychological distancing. Finally, forth are the set of functions facilitating the output of model data for further analysis, including post-experiment scripts

to parse model outputs and analyze results across multiple runs. All event scheduling in the model follows a Last-In-First-Out (LIFO) protocol.

The process and scheduling of biosecurity decision-making (investments and compliance) follow the schematic of Figure 2.

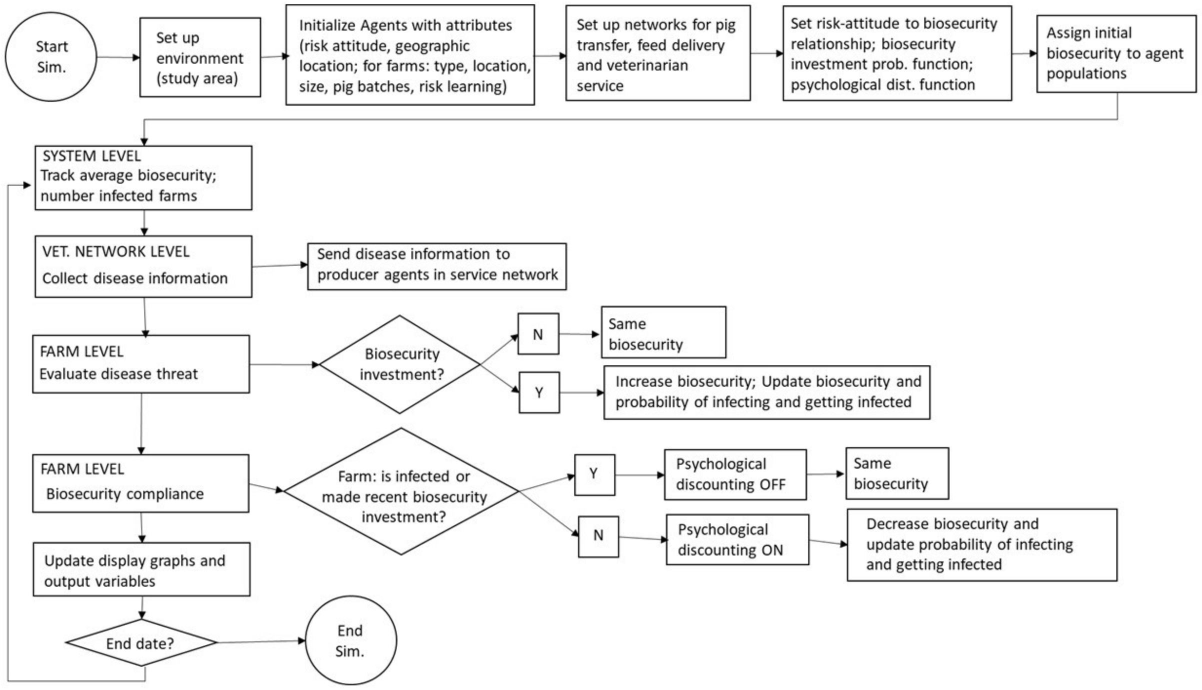

Figure 2: Schematic of decision-making for biosecurity investments and biosecurity compliance.

II) Design Concepts

II.i Theoretical and Empirical Background

II.i.a Which general concepts, theories or hypotheses are underlying the model's design at the system level or at the level(s) of the submodel(s) (apart from the decision model)? What is the link to complexity and the purpose of the model?

Because real-world epidemics are fundamentally phenomena which propagate through networks (social, business, transportation, etc.), the formulation of a suitably-realistic network structure within which agents operate is a fundamental basic principle of the model. A corollary to this basic principle concerns the model's balance between context specificity and analytic transparency. The model's network generation algorithm strives to maintain sufficient context specificity to capture the critical complexities underpinning observed epidemiological spread phenomena, while bracketing superfluous elements of real-world production chain networks, which have not been implicated in previous epidemiological events. For example, the model contains only feed mill, producer, slaughter plant and

veterinarian agent typologies, because these were identified by industry experts as the critical players underpinning disease spread. Whereas in real-world hog production chain networks there may be a multitude of other actor typologies (i.e. equipment suppliers, construction contractors, insurance agents, and many more), these were intentionally excluded from the model's design to simplify analysis.

Another guiding principle is the geospatial situation of the model within real U.S. states. The states of North Carolina, Iowa, and Illinois were chosen because they produce a large number of the nation's hogs, as well as being amongst the most hog-dense states. In many epidemiological studies, agent density has been shown to impinge directly upon spread characteristics. With high enough density, complex phenomena such as percolation thresholds may emerge.

In the model, human risk attitude is at the base of the human behavior submodel. Even though there can be other influencing factors, we focused on risk attitude because we had data from our experimental games that we could use to both initialize and parameterize the model. Mechanisms simulating human behaviors are encoded for producer agents but not for feed mills or slaughter plants. Veterinarian agents are only responsible for sending messages to producers and do not take part on any dynamics happening in the production networks. Our behavioral functions are empirical. The shape of the relationships between risk attitude and biosecurity investment and between risk attitude and biosecurity compliance are built using general mathematical equations whose parameterization was achieved with a calibration experiment. The risk-attitude learning mechanism is agnostic meaning that the producers can change their attitude according to rules derived statistically from experimental games and assigned to agents at model start. The learning is not generated contextually in the simulation as a response to interactions with the model environment.

Agents in our model are distinguished by several attributes, some of which describe and drive human decision-making and behavior in relation to biosecurity, namely, risk attitude, learned risk strategy, psychological distancing, and biosecurity increase and disease-response parameters for the function modeling the probability of deciding to increase biosecurity. The combination of attributes risk attitude and learned risk strategy creates the behavioral identity of the producer and drives the biosecurity values in the simulated environment. In particular, the producer agents have the ability to respond to their environment represented in our ABM by the epidemiological conditions. They receive epidemiological information in messages sent by their veterinarian agent. In general our producer agents exhibit the following characteristics:

- Autonomous**:** They exercise control over their own actions.
- Social**:** they communicate and interact with other agents.
- Reactive: they respond to external cues.

The feed mills and slaughter plants agents are only characterized by an initial risk attitude that determines their biosecurity value. They do not have the capacity to sense and respond to their environment and they also do not have encoded learning. This means that their biosecurity is set and remains fixed throughout the simulation. The veterinarian agent is external to the system and only present to send information on disease level to producers. Therefore it does not have any behavioral attributes.

In our model, attitude towards risk refers to a person's positive (favorable) or negative (unfavorable) evaluation of taking risk when there is a disease outbreak. For example a risk tolerant is an individual (or a model agent) who sees tolerating risk coming with some benefit such as saving biosecurity expenditures and therefore higher income. On the other end, a risk averse individual (or agent) holds a

negative risk attitude meaning that he/she sees risk as harmful to their business. We considered risk attitude as a predictor of biosecurity behavior. Specifically, an individual's attitude to tolerate or avoid risks has an effect on decision-making (and biosecurity behavior) under conditions of disease risk. In our model, risk attitude affects the probability to invest in biosecurity and the level of compliance kept with biosecurity protocols. We recognize that risk attitude can be situational. Therefore, we collected data on attitude from experimental games where the participants were presented with a situation similar to the ABM environment where they had to make a decision on whether or not to invest in biosecurity given a probability of disease contagion in their farming area.

II.i.b On what assumptions is/are the agents' decision model(s) based?

The primary set of assumptions driving agent behavior relate to trade patterns associated with the industry role each agent plays, with agents in the model operating in accordance with general industry norms. For example, it is assumed that, as soon as their livestock batches reach the transfer age appropriate for their industry role, producer agents will search the agent space for trading partners until an appropriate partner is found, at which point the pig batch will be immediately transferred.

Another assumption concerns the spatial locations of non-producer agents. Since fine-grained spatial data were not available—and the FLAPS tool only covers livestock production units (Burdett et al. 2015)—locations of non-producer agents are initialized by distributing them at random positions within each county, in proportion to the number of producers in the county.

Several assumptions also come into play concerning the distance, similarity, and number of other industry actors with which each agent may interact. These assumptions were parameterized using the nearest neighbor network structure.

A further assumption is our representation of livestock in batches (or metapopulations) of animals of the same age. This was primarily done to reduce computational overhead (vs. storing each animal's parameters individually). In addition to having the same theoretical birthday, it is assumed that if a batch is infected, all of its members are infected.

Finally, it is assumed that if a producer agent becomes infected, all of its livestock batches become infected. While a simplifying assumption to be sure, this is reasonably realistic, owing to the high observed virulence of the PED virus, which tends to sweep quickly through entire herds.

II.i.c Why is/are certain decision model(s) chosen?

Agents' decision heuristics primarily relate to their day-to-day operations, and rely on parameters including risk attitude, information access, industry role, size, and spatial location. Based on industry standards along with individual parameters, agents make decisions pertaining to when and with whom inter-agent contact will occur as well as decisions on biosecurity investment and compliance. The agent contact patterns go on to impact the susceptible / infective state variable of each agent, according to probabilities associated with each modality of inter-agent contact. Biosecurity-related decisions directly affect the vulnerability to disease both as the probability of becoming infected or passing infection during agent contacts.

II.i.d If the model/submodel (e.g. the decision model) is based on empirical data, where do the data come from?

We use the Farm Location and Agricultural Production Simulator (FLAPS) tool—which draws upon USDA Census of Agriculture data along with aerial imaging to impute realistic distributions of livestock farms within a specified U.S. region—to set producer agent locations and key operational parameters including industry roles and capacities (Burdett et al. 2015). While the FLAPS tool serves as our primary means to set production unit locations and operational parameters, our team also gained access to internal records from a large family-owned hog production chain system—identified as "FHPC" in Table *7*—which was used to impute realistic contact rate and shipment size parameters. Several other sources of empirical data were also used to parameterize the model, also indicated in *Table 7* and *Table 8*.

We assigned the risk-attitude attribute to the agent populations based on the distribution found by Clark et al. (2020). This distribution were derived from the analysis of data collected with an experimental game where participants were asked to make biosecurity investments under different scenarios of disease contagion, disease information availability and uncertainty. The groups resulted from a K-means algprithm based on a risk aversion rating calculated with experimental game data. The groups are: risk averse (invest in biosecurity both at high and low contagion rate); risk tolerant (invest very little in biosecurity at either low or high contagion rate) and; risk opportunist (invest in biosecurity when the contagion is high but not much when the contagion is low). This risk-propensity distributions are used as input to assign risk propensity in the ABM agent populations. The data from the same experimental game were used to parameterize risk-attitude learning.

II.i.e At which level of aggregation were the data available?

The FLAPS system uses several core datasets to impute producer agent locations and operational characteristics. The system primarily relies upon the 2012 USDA Census of Agriculture, which is aggregated at the county level. However, FLAPS also leverages aerial imaging land use data to position agents more precisely within each county. Slaughter plant data from USDA NASS (2014) were aggregated at the level of the U.S. state.

II.ii Individual Decision-Making

II.ii.a What are the subjects and objects of the decision-making? On which level of aggregation is decision-making modelled? Are multiple levels of decision making included?

Decision-making about the transfer of pigs and feed deliveries is modeled at the level of the agent, be it a producer, feed mill, or slaughter plant. When transferring livestock, for example, a producer agent will wait until a pig batch matches the appropriate age corresponding to its industry role. This subject will then search among its potential trading partners (objects, in this case), which were pre-selected at model initialization to be of the appropriate industry role, until a suitable agent is found that has sufficient capacity to accept the shipment.

Decision-making about biosecurity investment and biosecurity compliance is modeled at the level of the producer agent following the schematic of figure . When receiving information on the network disease

status from the veterinarian agent, a producer agent will make a decision on whether to invest and increase biosecurity or remaining at the current level or not. The decision is probabilistic and depends on the agent's risk attitude and the information received from the veterinarian on disease status. The decision on biosecurity compliance is instead dependent on the disease status of the agent making the decision, its risk attitude and whether the agent has recently invested in new biosecurity (recent investments are assumed to motivate compliance).

II.ii.b What is the basic rationality behind agent decision-making in the model? Do agents pursue an explicit objective or have other success criteria?

Agents in the model act according to accepted industry operational standards. They do not pursue a specific "objective," per se, other than to efficiently take in new livestock and ship them out at the appropriate life cycle stage. The model incorporates adaptive agent decision-making, for example allowing for decisions which reduce an agent's vulnerability if a disease is present in the network.

II.ii.c How do agents make their decisions?

A series of cyclically-executing functions (outlined in the *Implementation Details* section) govern how and when agents make decisions. These are based on the industry role of each agent, its risk attitude and its disease status. Agent behavior is calibrated to reflect the decision-making heuristics of real-world hog producers, as identified through producer surveys and other data gathering efforts.

II.ii.d Do the agents adapt their behavior to changing endogenous and exogenous state variables? And if yes, how?

Producer agents desiring to transfer livestock to the next production phase adapt their behavior based on the operational variables associated with their potential trading partners. This primarily comes down to finding a partner within a certain distance, of the appropriate industry role, of sufficiently-similar size, and with sufficient excess capacity to accept the shipment. The number of pigs housed at each production unit is constantly updated as the model runs, so the agents have to perform this search with each new outgoing shipment. However, agents in the current model do not adapt their livestock-transfer decisions based on the prevalence of disease in the system, or other global factors.

Producer agents adapt their biosecure behavior based on disease presence on both their production site and in the network (endogenous state variables) and their risk attitude. This dynamic behavior relies on messages exchanged between the veterinarian agent and the producers and lead the producer agents to make decisions on biosecurity investments and compliance to protect its farm.

II.ii.e Do social norms or cultural values play a role in the decision-making process?

Agents follow basic industry standards when making their decisions, which could be considered a kind of social norm. Values do not play into their choices, however.

II.ii.f Do spatial aspects play a role in the decision process?

Producer agents looking to transfer animals to another producer are constrained by a maximum distance parameter. The service areas of slaughter plant and feed mill agents are governed by Poisson distributions, with producers most likely to connect to the λth-closest of each.

Producer agents make their biosecurity investment decisions based on information limited by the area covered by their veterinarian network. They do not have access to full knowledge on the number of infected producers in the whole simulated region.

II.ii.g Do temporal aspects play a role in the decision process?

Producer agents will only transfer livestock to their producer trading partners if the partner has sufficient excess capacity. Since the inventory of each agent is constantly in flux, the time when the transfer function is executed will determine the trading partner that is chosen. Farrowing producers will also wait to batch weaner pigs until the quantity of piglets is greater than or equal to the minimum batch size, as a proportion of their capacity.

There is a time lag (disease incubation time) between the infection event and the pig becoming symptomatic. During this time the disease can spread through the network if the pigs are transferred or a truck becomes infected by visiting the farm.

The risk-attitude learning mechanism takes place over the simulation time period and occurs in monthly time-steps. It is encoded by shifting the value of the parameter x_0 (Table 12) connected by risk attitude.

II.ii.h To which extent and how is uncertainty included in the agents' decision rules?

Agent behavior does not account for uncertainty in the current model. However, we are pursuing behavioral research which will be used to parameterize agents in future model versions to react to uncertainty as regards disease prevalence in the networks (Merrill et al. 2008).

<u>II.iii Learning</u>

II.iii.a Is individual learning included in the decision process? How do individuals change their decision rules over time as consequence of their experience?

A learning process is included in the model for the producer agents. However, this is not learning in the strict sense of learning about the state of the world and how best to behave in it. The learning is agnostic and consists in an update of the decision rules that govern the biosecurity investment probabilities. This change reflects an evolution in the producers' risk attitude. The learning is hard-coded to reproduce learning trajectories in risk attitude found in the data collected in our experimental games.

The agents' decision rules related to hog and feed transfers remain non-adaptive in the current model The agents' action heuristics related to hog transfer are based on their industry roles, and are designed to realistically replicate throughout in the production chain system as a whole. Thus, an agent will transfer hoofstock to an appropriate trading partner as soon as possible, farrowing will proceed regularly wherever a producer has sufficient excess capacity, and feed deliveries will take place at a set

frequency. Each agent will necessarily adapt to market conditions resulting from the available spare capacity of its trading partners.

The decision rules related to biosecurity investment and compliance reflect a reactive nature of the producer agents. The agents change their biosecurity in response to model conditions: the presence of a disease within the network (biosecurity investments) and their own infection status (biosecurity compliance).

II.iii.b Is collective learning implemented in the model?

No.

<u>II.iv Individual Sensing</u>

II.iv.a What endogenous and exogenous state variables are individuals assumed to sense and consider in their decisions? Is the sensing process erroneous?

Producer agents “sense” the operational variables associated with potential trading partners when making decisions concerning livestock transfers. An agent’s perception of these factors is not erroneous, as it is based on a direct query of the potential recipients’ operational variables at the time the transfer is desired.

II.iv.b What state variables of which other individuals can an individual perceive? Is the sensing process erroneous?

The primary state variables agents perceive when making livestock transfer decisions are the potential trading partner’s industry role, capacity, inventory, and proximity.

Agents are programmed to sense external disease conditions around them via messages sent by the veterinarian agents.

II.iv.c What is the spatial scale of sensing?

At model initialization, producer agents generate a list of potential producer trading partners only within a given distance (150 km). It could be said that their “sensing” of other producers does not extend beyond this distance. Note that (based on the Poisson distributions discussed above), producers may very well interact with slaughter plants and feed mills beyond the 150-km limit imposed upon producer to producer transfers.

During the run, the producer agents sense (receive information about) disease presence in the area covered by their veterinarian agent’s service network. The area’s spatial extent can vary from run to run as the veterinarian are randomly located in the study area and each producer is connected to their nearest veterinarian agent. The sensing distance also depends on the number of veterinarians programmed at initialization.

II.iv.d Are the mechanisms by which agents obtain information modelled explicitly, or are individuals simply assumed to know these variables?

The mechanisms are modeled explicitly with the presence of veterinarian agents that collect disease information and send it throughout the network of producers via weekly messages.

II.iv.e Are the costs for cognition and the costs for gathering information explicitly included in the model?

No.

II.v Individual Prediction

DITTO ABOVE FOR 'PERCEPTION'

II.v.a Which data do the agents use to predict future conditions?

Agents do not currently engage in predictive behavior concerning the likely outcomes of their actions.

II.v.b What internal models are agents assumed to use to estimate future conditions or consequences of their decisions?

II.v.c Might agents be erroneous in the prediction process, and how is it implemented?

II.vi Interaction

…AND 'INTERACTION'

II.vi.a Are interactions among agents and entities assumed as direct or indirect?

II.vi.b On what do the interactions depend?

II.vi.c If the interactions involve communication, how are such communications represented?

II.vi.d If a coordination network exists, how does it affect the agent behavior? Is the structure of the network imposed or emergent?

II.vii Collectives

II.vii.a Do the individuals form or belong to aggregations that affect and are affected by the individuals? Are these aggregations imposed by the modeler or do they emerge during the simulation?

Livestock in the model may be considered as collectives, as they are encoded in groups of animals of the same age, and with the same infectivity status. If a producer is infected, it is assumed that all individuals in all pig groups owned by that producer will become infected.

In addition, while not defined explicitly as such, groups of agents in the model exhibit emergent collective characteristics due to their differential spatial distribution across the model's GIS space. For example, in densely-packed areas, groups of agents tend to interact heavily within connected clusters, leading to localized disease outbreaks. This type of emergent collective behavior is not directly imposed

by the modeler, although the fixed spatial location and network structure that is imposed impacts how and where this emergent phenomenon arises.

Producer agents belong to a veterinarian network that is imposed by the modeler and set at model inception. The veterinarian network affects the information content (number of infected premises in the network) that the producer receives.

II.vii.b How are collectives represented?

They represented with networks.

<u>II.viii Heterogeneity</u>

II.viii.a Are the agents heterogeneous? If yes, which state variables and/or processes differ between the agents?

As described in the *Entities, state variables, and scales* section above, agents fall into three main categories: (a) producers, (b) slaughter plants, and (c) feed mills. Producer agents are assigned one of six industry roles based on the USDA classification system for hog producers. An agent's industry role determines the initial age of its hoofstock, its hoofstock age transfer condition, as well as the appropriate trading partners, which make up its latent link set. These relationships are visualized in Figure 1.

Agents are also heterogeneous with respect to their probabilities of spreading or receiving an infection. These differential probabilities are set at model initialization based on the infection-spread risk laid out in Table 9 and Table 10.

II.viii.b Are the agents heterogeneous in their decision-making? If yes, which decision models or decision objects differ between the agents?

Agents' decision-making will differ depending upon their industry role. For example, a farrow-to-wean producer will only send pigs to wean-to-feeder or wean-to-finish producers. Their decision-making is also differing based on the risk attitude. For example, a risk averse agent has a higher probability to make a biosecurity investment than a risk tolerant agent given the same disease-status in their veterinarian network.

<u>II.ix Stochasticity</u>

II.ix.a What processes (including initialization) are modelled by assuming they are random or partly random?

RUSHPCBM uses both fixed-seed stochasticity (for initialization) as well as random-seed stochasticity (controlling infection spread). All initialization procedures utilize a seed in all draws from stochastic functions that is fixed across all model runs in an experiment. This is important because we are interested in analyzing the dynamics associated with disease risk, and changing the basic supply chain network structure across runs would confound results.

Firstly, the fixed seed is used to initialize several agent parameters that are not directly read in from the database—i.e. producers' all-in-all-out status and the list of potential transferees for each agent, both of which are drawn using a uniform distribution.

We also use fixed-seed draws from custom distributions—i.e., those based on the number of observations in a series of categories—for two initialization tasks, these being the setting of agents' biosecurity levels, risk attitude, and risk-attitude learning trajectory as well as the spatial location of slaughter plants, and feed mills to correspond with producer density by county.

Once initialized as described above, the remainder of the stochasticity in the model uses a random seed, allowing us to analyze a distribution of infection and contact networks resulting from a series of model runs. Random-seeded stochasticity is first used to populate each producer's initial pig batches. Both the size and age of the pig groups associated with each producer are drawn from a uniform distribution bounded according to the producer's industry role. Since each producer will always start with a slightly different animal inventory, this ensures that the trade patterns that unfold throughout each model run are not repeated exactly.

Random-seed stochasticity is also used for all disease-spread calculations. Thus, whereas a given agent's disease spread probability values stay constant across runs, the result of any given random draw using these probabilities is always random. Uniform probability distributions returning "true" if a randomly-drawn value between zero and one is less than p are used to determine if infection will spreads.

Finally, random-seeded Poisson distributions are used to determine the number of producers to visit for each feed distribution trip.

II.x Observation

II.x.a What data are collected from the ABM for testing, understanding and analyzing it and how and when are they collected?

The model tracks in real-time the current hoofstock inventory of all producers in the model, the number of currently infected hoofstock, the number of currently infected producers, and the cumulative number of infected producers, which can be output as time-series data to examine infection-spread dynamics.

In addition, we track the flow of feed and livestock between different types of agents in order to calibrate model parameters to reflect real-world data, for example the distribution of hog shipment sizes and delivery frequencies characteristic to real hog supply chain networks.

Finally, a contact network adjacency matrix with link weights encoding the number of times each agent interacted throughout the model run is exported as tabular data after each run, and later parsed using a series of Python functions. An infection-spreading network is similarly tracked, output, and parsed. Key statistics on trade and infectivity patterns across a series of model runs—both at the individual agent as well as the whole-network level—may then be analyzed.

II.x.b What key results, outputs or characteristics of the model are emerging from the individuals? (Emergence)

Emergent phenomena in the present model will occur as a result of the structural elements of the model, for example the network configuration and disease spread characteristics specified by the user. This could take the form of differential spread characteristics resulting from user-input parameters concerning disease spread probabilities and durations, such as in the observation of percolation thresholds.

III) Details

III.i Implementation Details

III.i.a How has the model been implemented?

The model was implemented using AnyLogic v.8.7 software, which relies upon the Java programming language for all scripts and functions. The sections below use pseudocode to describe in detail the algorithmic structures underlying each model function.

- Infection

Porcine epidemic diarrhea virus (PEDv) can be transferred from one ABM agent to another on the network connections used for animal and feed movement. Associated with each movement there is a probability to transmit or contract infection dependent on the type of movement and the biosecurity of the interacting agents. The agent interactions are mediated by a theoretical truck, both in the case of feed deliveries, and of pig movements among producers or between a producer and a slaughter plant. The probability of infection for each type of interaction is calculated using an independent logistic equation multiplied by a seasonal adjustment. We model the logistic functions to reflect the fact that agents are less likely to become infected when more biosecurity measures are taken to prevent virus incursion. Because PEDv is a seasonal virus with highest infection risk in winter months, we add a seasonal adjustment that modulates the infection probability with time. The probability of infection is given by the following function:

$$p_{infection} = adj_{season} \cdot p_{logistic}$$

Where the logistic probability $p_{logistic}$ and the seasonal adjustment adj_{season} are defined by:

- The logistic function:

$$p_{logistic} = (p_{max} - p_{min}) \cdot \frac{1}{1 + e^{m \cdot (bs - bs_0)}} + p_{min}$$

- and the sinusoidal function:

$$adj_{season} = (1 - min_{adj}) \cdot \frac{1}{2}(1 + \cos(2\pi \cdot \frac{d - d_{peak}}{366})) + min_{adj}$$

and the parameters are described in Table 10. For parsimony in the model's parameter values, we set p_{min}=0.05, m=1.3, and bs_0 = 4 for all agent interactions. We then estimated the logistic's p_{max} to fit the

probability estimates provided by field experts for different types of interactions between hog production premises, feed mills and slaughter plants with a medium biosecurity during the high infection months of winter (Table 10 and Table 11). The seasonality adjustment function adj_{season} oscillates between a maximum $max_{adj} = 1$ reached at day $d_{peak} = 30$ (January 30th) and a minimum $min_{adj} = 0.3$ in the summer. Figure 3 displays the shape of the probability of infection function through time and across the biosecurity gradient in 3D.

Table 10: Parameters of the probability of infection function including both the logistic and the sinusoidal forms.

**Symbol**	**Infection probability function's term**	**Definition**	**Description**
bs		Input biosecurity	Continuous variable describing an agent's biosecurity over the range [0, 8].
p_{max}	$p_{logistic}$	Carrying capacity	$p_{max} \leq 1$<br>A set maximum value for the probability (i.e. what happens at high/infinite biosecurity).
p_{min}	$p_{logistic}$	Minimum value	Probability value at biosecurity = 0
m	$p_{logistic}$	Slope of the curve	Parameter is related to the steepness of the curve at point x0. Note that m < 0 to have a Z-shaped function
bs_0	$p_{logistic}$	Point of inflection	The point on the Z-shaped curve halfway between *minf* and *K* (i.e. the center of the logistic curve).
d	adj_{season}	Input day of the year	Current day of the year [1, 365] or [1, 366] for leap years
d_{peak}	adj_{season}	Day with highest infection (peak)	Day of the year at which the adjustment is equal to 1.
min_{adj}	adj_{season}	Scaling parameter	Parameter to keep the seasonal adjustment bounded within [min_{adj},1]

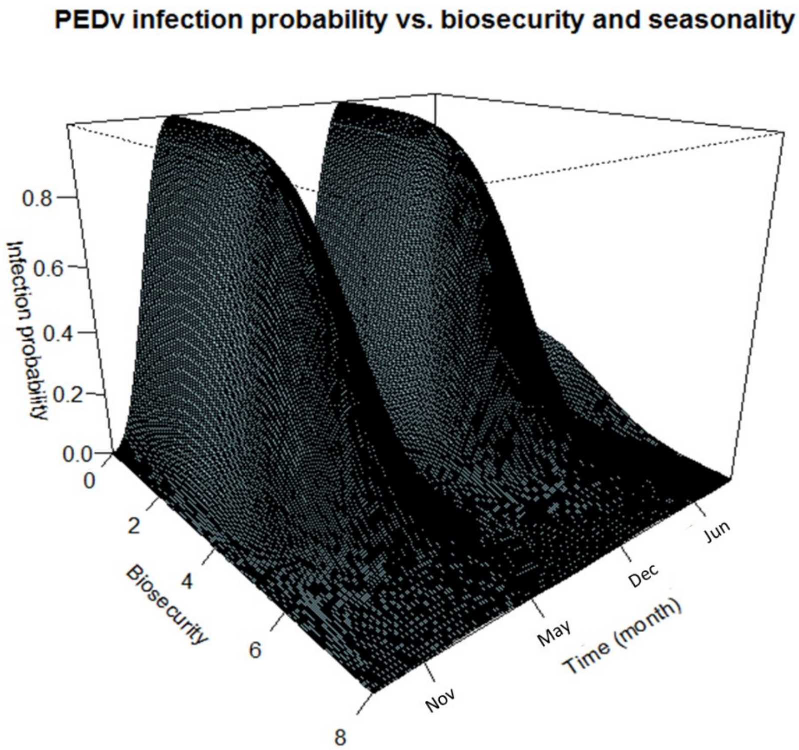

Figure 3**:** General logistic shape of the PEDv infection probability function used in the ABM. The probability of infection increases with decreasing values of biosecurity. The sinusoidal variability through time accounts for PEDv seasonality with maxima in winter (January) and minima in summer (June).

Table 11: Parameters for each agent interaction that can carry infection. From logistic function with four parameters (p_{max}, p_{min}, m, bs_0) of probability of PEDv transmission: parameters p_{max} (column 2), p_{min}=0.05, m=1.3, and bs_0 = 4 provides the probability of infection when biosecurity=4 on January 30 for all the probability functions. The only exception is p_{min}=0.005 in the probability function for the infection from visitors at the producers sites (last raw).

**Infection probability**	**Infection probability parameter p_{max}**
Prob. producer will become infected if returning pig truck is contaminated	0.35
Prob. producer will become infected if feed truck is contaminated	0.8
Prob. feed truck will become contaminated if producer is infected	0.15
Prob. pig truck will become contaminated if producer is infected	0.4

Prob. feed mill will become infected if returning feed truck is contaminated	0.25
Prob. feed truck will become contaminated if feed mill is infected	0.99
Prob. slaughter plant receiving area will become infected if pig batch is infected	0.99
Prob. pig truck will become contaminated if receiving area is infected	0.25
Prob. producer will become infected if visitor truck if is infected	0.008

- Responsiveness and biosecurity investment

The agent-based model is used to model realistic socio-structural processes by including social, cognitive, and organizational models. It also explicitly represents spatiotemporal elements of the agents and the environment. This then allows for the modeling of the transition between standard operations of the hog industry and operations under an outbreak. The hog production system mostly operates following standard procedures/operations and the transition from these standard procedures to the response to the presence of a disease in the large system a key aspect of biosecurity.

The ABM agents start with a set of predefined stimulus-response rules controlled by the risk attitude (Figure 4). In our ABM, the farm agent's intention to invest in biosecurity or not is influenced by two factors: the agent's risk attitude and the information on disease available to the agents. The way in which information is transmitted is by having each producer agent connected with a veterinarian agent, who collects information about disease occurrence in its farm service network and reports the number of infected farms weekly across the network. Reflecting the results obtained in our experimental game, we model the probability of investing in biosecurity and the number of infected producer agents in the veterinarian's network as directly related. The probability that a producer agent make a biosecurity investment upon receiving the veterinarian's message is described by a logistic function dependent on the number of farms infected in the agent's environment (Figure 4):

$$p_{logistic} = (p_{max} - p_{min}) \cdot \frac{1}{1 + e^{m \cdot (NI - rs * NI_0)}} + p_{min}$$

where *NI* is the number of infected producers in the veterinarian's network where the agent belongs, the parameter NI_0 is the number of infected producers at the inflection point, p_{max} and p_{min} are the minimum and maximum probability values, m is the steepness of the curve and *rs* controls the relative distance among the probability curves (NI_0) describing the biosecurity increase for the three risk categories (Figure 4). We refer to NI_0 * *rs* as the parameter x_0. The parameter values of the logistic function vary for each of the three possible risk attitude categories (Table 12). The curves embody risk preferences as well as other factors (in our case the number of infections in the network) that influence

an agent's intention to invest in biosecurity. For example, agents in the risk averse category increase biosecurity more promptly in response to disease presence than agents in the risk tolerant category, who instead probabilistically start to increase biosecurity at higher infection numbers (Figure 4). In the model, the probability curves are converted to behavior by drawing a random value from a standard uniform distribution. If the random number is smaller than the probability to increase biosecurity, the producer agent increases its biosecurity level by a fixed quantity; otherwise, the agent maintains its current biosecurity level.

Table 12: Parameters of the biosecurity-investment logistic function for each of the three risk categories (risk averse, risk opportunist, and risk tolerant). The x_0 values were estimated with a calibration experiment matching observed and simulated PEDv incidence and kept fixed for the scenario experiment presented in this study.

**Logistic probability function**	**Risk attitude categories**		
**Parameter**	**Risk averse**	**Risk opportunist**	**Risk tolerant**
p_{max}	1	1	1
p_{min}	0	0	0
m	-0.95	-0.95	-0.95
NI_0	2	25	48
$x_0 = NI_0 * rs$ (rs = 0.07)	0.14	1.75	3.36

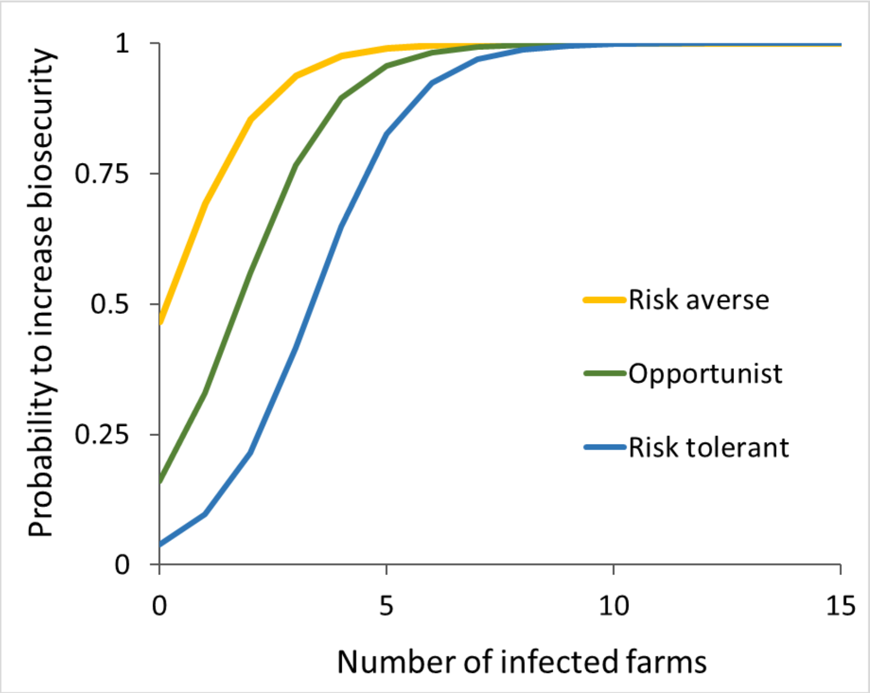

Figure 4: Logistic functions of the probability of biosecurity investment. The agent's probability to increase (or not) biosecurity is associated with the information about disease presence measured by the number of producer agents (farms) infected within the agent's veterinarian network. Once a week, the veterinarian agent communicates the disease information across its network of producers. In each communication event, a producer responds (or not) with an increase of biosecurity according to the logistic probability designed for the producer's risk attitude. The risk attitude influences the delay of response to the disease information as shown by the colored coded lines. With a relative shift parameter *rs*=0.07, risk averse producers have almost 50% probability of increasing biosecurity even when there is no infection in their veterinarian's network and their response reaches 99% at 5 infected farms. The 99% probability trigger for risk tolerant is 9 infected farms. Risk opportunist's response is similar to risk tolerant producers when the infections are few but shifts to be similar to risk averse producers as the number of infections increases.

- Psychological discounting and relaxation of biosecurity compliance

A relevant element of this model is psychological discounting, whereby the potential of maintaining an adopted biosecurity level is discounted through time with decreasing biosecurity compliance. Psychological discounting describes a phenomenon in which humans will underestimate risks progressively more when repeatedly participating in risk without experiencing negative consequences. If negative feedback such as disease incursions lags, human negligence intensifies and results in weak biosecurity compliance. Concretely, the approach assumes that as time passes without infections on the agent's farm, the perception of risk of infection and thus and biosecurity compliance decrease. The functional form that we adopted for such discounting is linear with time:

$$B_t = (1 - D) \cdot B_{t-1}$$

With B_t denoting the biosecurity level of a producer agent at time *t* and *D* the fixed discounting rate. The discounting process is only active during times when the agent is the susceptible state to reflect the situation in which the absence of disease leads to a relaxation in biosecurity compliance. Instead, during time when the agent is infected, we assumed high compliance with biosecurity protocols and the value of the parameter *D* in the model is set to 0. The parameter *D* = -0.001 and was estimated by model calibration (Table 15).

- Risk behavioral learning

To account for the fact that risk attitude can be influenced by experience, we included a learning process in our ABM. This learning process is not yet artificial intelligence. The agent learning is pre-set and encoded with a state transition matrix that contains the probabilities that an agent transitions from a risk attitude group to another over the course of a simulation. We call "learned risk strategies" the risk attitude resulting from these transitions. The transition probability values were derived from the experimental game that we run to study behavioral attitudes towards risk of infection (Clark et al., 2020). The analysis of participants' risk choices showed patterns of learning across the game scenarios and provided evidence for changes in risk attitudes (Figure 5).

The risk learning trajectories are agnostic and not related or triggered by simulation events. The agents evolve in their risk attitude according to the state transition matrix that represents a general experiential evolution of risk attitude and is independent of any specific event in the simulation. In this version of our ABM, the agents are therefore not fully intelligent. The learning process rests on the responses provided by the experimental game's participants. We assume that the encoded learning features comprise the participants' general learning from the experience of events featured in the experimental games such as direct experience of infection, disease vicinity, access to disease risk information and/or neighbors' biosecurity level information, risk uncertainty. We recorded their decisions during the gameplay, analyzed the data and extracted trajectories of change in risk behavior (Figure 5). These trajectories along with the relative fraction of participants following each trajectory (Figure 6) provided the base to calculate a risk-attitude transition probability matrix (Table 13) that provided relative proportion of people who followed each specific risk-attitude change.

The analysis of the gameplay data sought overall trends of risk attitudinal changes during the gameplay:

1. Learning: change from one initial attitudinal position (averse or tolerant) to the opposite attitudinal position. Learning included cases in which there was an attitudinal change in at least one of the two contagion scenarios, low or high.
2. No learning: same risk strategy during the whole gameplay.

In order to communicate easily and efficiently in this paper, we will use the simplified term "attitude learning trajectories" even when there was no actual learning. The gameplay schematic in Figure 5 shows the trends of all the possible combinations of trajectories for both scenarios of low and high contagion rates used in the experimental game.

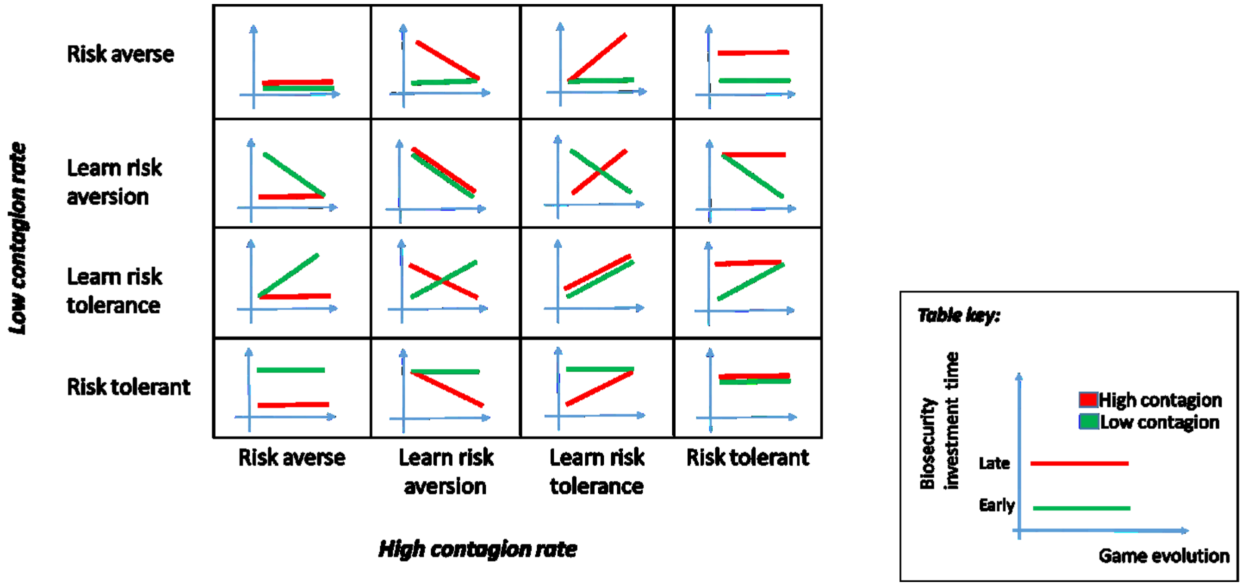

Figure 5: Table reporting trends of risk learning trajectories exhibited by the participants in the experimental game. Each game has several rounds with scenarios of either high or low contagion rates. The participants were asked to make decisions on biosecurity investments. Biosecurity investments made early in a round characterized risk averse attitudes while late biosecurity investments defined a risk tolerant attitude (last minute decision to build safety). Some game participants exhibited a learning process that lead to more risk averse or more risk tolerant attitudes as the game rounds progressed (x axis). Other participants did not exhibit any shift from their original attitude (no learning). Red lines exemplify the direction of learning trends in high contagion scenarios; green lines exemplify the direction of learning trends in low contagion scenarios. The horizontal axis in each graph represents the evolution of the game rounds. The vertical axis represents the time chosen by the participants for biosecurity investment within each round (some people are early biosecurity adopters while others are late adopters). The cases in which there is a different behavior for high and low contagion define an opportunist attitude: the participant changed risk attitude. Learning risk trajectories include cases of actual learning: graphs showing at least one positive or negative trend. Flat learning trajectories indicate cases in which the participant kept the same risk strategy across the gameplay (see the four corner cells in the matrix).

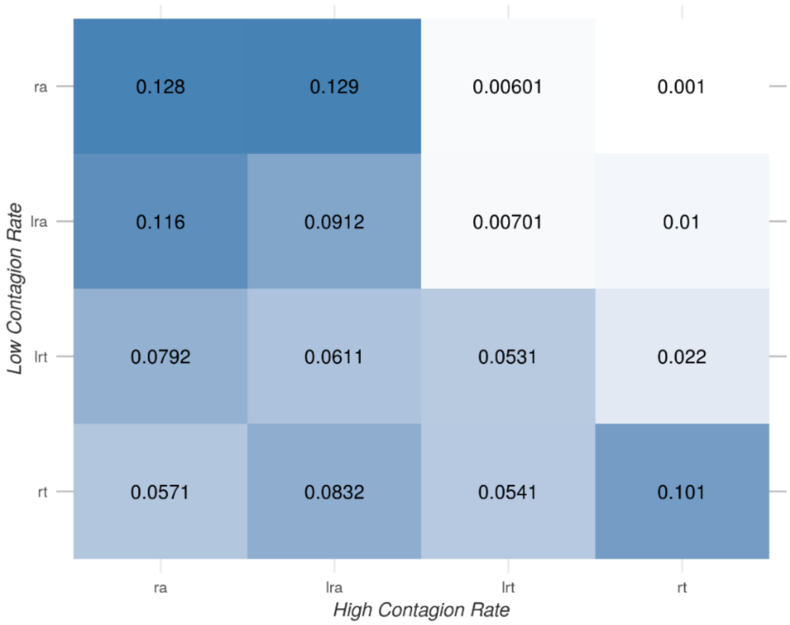

Figure 6: Table reporting the relative proportion of risk strategy behaviors in the participant sample at high and low contagion rates. Each cell reports the relative proportion of participants who exhibited the risk behavior shown in the table of Figure 5. The table labels are the same as in figure 6: “ra” stands for “risk averse; “lra” for “learn risk aversion”; “lrt” for learn risk tolerance” and ; “rt” for “risk tolerant”. The blues shades reflect the distribution proportions with darker blue shades indicating higher proportion values.

To link the three classes of risk attitude to learning behaviors (Figure 5 and Figure 6), we built a transition probability table (Table 13). The learning process was implemented by changing the shape of the biosecurity investment curves. Based on the transition probability matrix (Table 13), there are nine learning trajectories. A change in risk attitude is reflected as a change of the value of the parameter x_0 of the biosecurity-investment probability curve (Figure 4 and Table 12). The curve shifts if there a risk attitude change. The x_0 values changes in monthly steps such as to transform the curve designed for the initial attitude to match the curve designed for the final risk attitude by the end of the simulation. For example, the learning trajectory from risk averse to risk opportunist entails a change in the parameter x_0 from 0.14 to 1.75.

Table 13: Transition probability matrix for producer agents’ learned risk strategies. The first line in each cell reports the transition probability and the second line the cell row(s) and column(s) of the table in Figure 6 used to calculate the probability.

	**Final risk attitude**		
**Initial risk attitude**	Risk tolerant	Risk opportunist	Risk averse
Risk tolerant	0.1<br>(4,4)	0.09<br>[(4,2) + (2,4)]	0.09<br>(2,2)

Risk opportunist	0.07<br>[(4,3) + (3,4)]	0.12<br>[(4,1) + (3,2)]	0.25<br>[(2,1) + (1,2)]
Risk averse	0.05<br>(3,3)	0.08<br>[(3,1) + (1,3)]	0.13<br>(1,1)

III.i.b Is the model accessible, and if so where?

While the source code for the model is not accessible due to limitations of the AnyLogic software, the pseudocode below explicates the code at a high level of detail. In principle, these pseudocode functions may be used to implement the model using any desired programming language.

Notes on pseudocode used in this document:

- The characters "//" will be used to designate a descriptive comment (i.e., the line of text following the "//" is not part of the actual function logic).
- Parameters referenced in all functions refer to those associated with the agent object from which a function has been called. In some cases, to disambiguate, the terms "self" or "my" may be used to refer to the function-calling agent object or its associated parameters.
- "ADD OR INCREMENT [sender] in [receiver]'s [network edge list]" is defined here as:

```
IF [sender] is not in [receiver]'s [network edge list] ADD [sender] to
      [receiver]'s [network edge list] with contact counter set to 1
ELSE INCREMENT contact counter associated with [sender] in [receiver]'s [network
      edge list] by 1
```

- "RANDOM DRAW using [probability]" is defined here as the Boolean value resulting from:

```
(DRAW random number from uniform distribution between 0 and 1) < [probability]
```

III.ii Initialization

III.ii.a What is the initial state of the model world, i.e. at time t = 0 of a simulation run?

The model is initialized by progressing through a series of initialization functions. Several agent parameters are set as each agent object is generated by the model. Next, further agent parameters are set by reference to the model database. Finally, upon completion of the preceding, producer agents initialize their networks of potential trading partners (feed mills and slaughter plants) and the connection to the nearest veterinarian agent to be referenced throughout the model run.

- Initialization functions called from main object (in order of function calls):

Initialize map view function:

```
SET GIS map boundaries and zoom on U.I. dashboard to correspond to study area
```

Initialize agents function:

Numbers, locations, and typological distributions of producer agents within the model are generated heuristically using the Farm Location and Agricultural Production Simulator (FLAPS) system developed through a collaboration between Colorado State University and the United States Department of Agriculture (Burdett et al. 2015). FLAPS parses USDA National Agricultural Statistics Service (USDA NASS) databases along with land use data to impute spatially-explicit datasets depicting the distribution of livestock production units throughout the desired study area. Thus, while not representing actual farm locations, the producer agents in the model are distributed geographically and with characteristics including production volume and classification category in such a way as to be consistent with real-world distributions.

An implicit assumption we have made is that the distribution of slaughter plants and feed mills mirrors the distribution of producers by county. Once a non-producer agent's county has been assigned by drawing from this distribution, the agent is placed at a random set of coordinates within the selected county. Thus, counties with higher producer density will tend to have higher numbers of non-producer industry actors as well.

```
FOR EACH producer agent
   // read in data for each agent from database and set appropriately
   READ latitude and longitude from database table corresponding to study area
   SET agent's spatial location
   READ total capacity from database table corresponding to study area
   SET agent's total capacity
   READ producer industry role from database table corresponding to study area
   // in consultation with industry experts, producers classified as "other" in
         the USDA NASS data are assumed to be "wean to feeder"
   IF (database query result = "other")
      SET agent's industry role to "wean to finish"
   ELSE
      SET agent's industry role to correspond with database query result
   SET agent's icon color to match producer type
   IF (agent is a farrowing type)
      SET agent's sow inventory to MAX of 1 and (total capacity * sow ratio
            parameter appropriate for agent's type)
   ELSE
      SET agent's sow inventory to zero
   SET agent's non-sow capacity to (total capacity – sow inventory)


   // iteratively generate initial pig batches
   SET minimum batch size to (agent's total capacity * minimum batch size as
         proportion of capacity parameter)
   WHILE (minimum batch size < remaining capacity)
      IF (agent's total capacity <= capacity under which a producer is assumed to
            have only one batch parameter)
```

```
            SET batch size to agent’s non-sow capacity
        ELSE
            SET batch size to a random integer between minimum batch size and non-
                    sow capacity
            IF ((batch size + current pig inventory) > non-sow capacity)
                SET batch size to minimum batch size
        SET batch birthday to a random integer between the maximum and minimum age
                of a pig for the agent’s industry role
        ADD batch size and birthday to pig tracker
        INCREMENT agent’s current inventory by batch size


// distribute other agents by county to correspond with producer density
FOR EACH county in study area
    READ number of producers in county from database
    ADD county name to county distribution array as many times as there are
            producers in that county
FOR EACH slaughter plant agent
    DRAW RANDOM county name from county distribution array
    SET agent’s spatial location to a random point inside the county drawn
REPEAT above FOR loop for feed mill agents


// now that locations and characteristics are set, run each producer’s network
        initialization function in turn
FOR EACH producer agent
    CALL agent’s “initialize network” function
```

- Initialization functions executed upon agent object creation:

Compute infection probability functions:

```
DRAW biosecurity level from biosecurity distribution customized for each risk
        attitude group
FOR EACH infection spread probability associated with the agent’s classification
    SET logistic function parameters
```

- Initialization functions called from main object (in order of function calls):

Initialize map view function:

```
SET GIS map boundaries and zoom on U.I. dashboard to correspond to study area
```

Initialize agents function:

Numbers, locations, and typological distributions of producer agents within the model are generated heuristically using the Farm Location and Agricultural Production Simulator (FLAPS) system developed through a collaboration between Colorado State University and the United States Department of Agriculture (USDA).[1] FLAPS parses USDA National Agricultural Statistics Service (USDA NASS) databases to generate spatially-explicit datasets depicting the distribution of poultry and livestock farms throughout the U.S. Thus, while not representing actual farm locations, the producer agents in the model are distributed geographically and with characteristics including production volume and classification category in such a way as to be consistent with real-world distributions.

An implicit assumption we have made is that the distribution of slaughter plants, auction houses, and feed mills mirrors the distribution of producers by county. Once a non-producer agent's county has been assigned by drawing from this distribution, the agent is placed at a random set of coordinates within the selected county. Thus, counties with higher producer density will tend to have higher numbers of non-producer industry actors as well.

```
FOR EACH producer agent
    // read in data for each agent from database and set appropriately
    READ latitude and longitude from database table corresponding to study area
    SET agent’s spatial location
    READ total capacity from database table corresponding to study area
    SET agent’s total capacity
    READ producer industry role from database table corresponding to study area
    // in consultation with industry experts, producers classified as “other” in
            the USDA NASS data are assumed to be “wean to feeder”
    IF (database query result = “other”)
            SET agent’s industry role to “wean to finish”
    ELSE
            SET agent’s industry role to correspond with database query result
    SET agent’s icon color to match producer type
    SET agent’s all-in-all-out status based on all-in-all-out probability global
            parameter associated with agent’s industry role
    SET agent’s sow inventory to MAX of 1 and (total capacity * sow ratio
            parameter appropriate for agent’s type)
    SET agent’s non-sow capacity to (total capacity – number of sows)


    // iteratively generate initial pig batches
    SET minimum batch size to (agent’s total capacity * minimum batch size as
            proportion of capacity parameter)
    WHILE (minimum batch size < remaining capacity)
        IF (agent’s total capacity <= capacity under which a producer is assumed to
                have only one batch parameter)
            SET batch size to agent’s non-sow capacity
        ELSE
```

[1] Available at: http://flaps.biology.colostate.edu/

```
        SET batch size to a random integer between minimum batch size and non-
            sow capacity
        IF ((batch size + current pig inventory) > non-sow capacity)
            SET batch size to minimum batch size
    SET batch birthday to a random integer between the maximum and minimum age
            of a pig for the agent's industry role
    ADD batch size and birthday to pig tracker
    INCREMENT agent's current inventory by batch size


    // distribute other agents by county to correspond with producer density
    FOR EACH county in study area
    READ number of producers in county from database
    ADD county name to county distribution array as many times as there are
            producers in that county
    FOR EACH slaughter plant agent
        DRAW RANDOM county name from county distribution array
        SET agent's spatial location to a random point inside the county drawn
    REPEAT above FOR loop for feed mill and then auction house agents


    // now that locations and characteristics are set, run each producer and
            auction house agent's network initialization function in turn
    FOR EACH producer and auction house agent
        CALL agent's "initialize network" function
```

- Producer agent initialize network function:

Once the agents' locations and industry roles have been initialized, a network initialization function generates a set of potential trading partners for each agent. All producer agents are assigned one feed mill and one auction house, and finishing producers are also assigned one slaughter plant, both connections being to the closest of that agent type. A pool of potential transferee producers is also generated for each producer according to their industry role. These relationships are shown in Figure 1. The potential transferee producers in this potential transferee pool are filtered according to (a) the maximum producer-to-producer connection distance parameter, as well as (b) the maximum number of transferee producers parameter.

```
Potential farms list = FILTER other producer agents s.t. (industry role of other
        producer is the next step in the production chain) AND (distance to the other
        producer <= max producer-producer connection distance global parameter))
WHILE (there are still potential farms AND (my transferee producers <= max number of
        transferee producers global parameter))
        DRAW RANDOM from potential farms list and ADD to my transferee producers
IF my transferee producers is empty
        ADD nearest farm of appropriate industry role
IF industry role is a finishing type
        SET my slaughter plant to the closest slaughter plant
SET my feed mill to the closest feed mill
```

III.ii.b Is the initialization always the same, or is it allowed to vary among simulations?

The initialization of the spatial location, operational characteristics, biosecurity levels, and potential trading partners for each agent remains consistent across runs within each of our three study area states. Thus, there are in essence three distinct initial states with regard to the above parameters, defined by the study areas. However, the initial livestock population housed at each producer premises differs between runs, as do the real-time trading choices and infection spread patterns experienced by each agent. For more detail, see the 'Stochasticity" section.

III.ii.c Are the initial values chosen arbitrarily or based on data?

Initialization parameters rely upon several datasets, including the University of Colorado / USDA FLAPS system, USDA NASS data, USDA APHIS data, Google Maps queries, and livestock industry datasets. For details, see the initialization function descriptions and pseudocode above.

<u>III.iii Input Data</u>

III.iii.a Does the model use input from external sources such as data files or other models to represent processes that change over time?

The model relies upon an external database to store many of the initialization values. Once set, these values remain static throughout each model run.

<u>III.iv Submodels</u>

III.iv.a What, in detail, are the submodels that represent the processes listed in 'Process Overview and Scheduling'?

- Producer agent cyclically-executing functions:

Farrow, wean, and batch piglets function:

If a farm which farrows piglets (Farrow to Wean, Farrow to Feeder, or Farrow to Finish types) is left with excess capacity after a livestock transfer, a farrowing function fills that capacity with a new batch of piglets, whose birthday is set to the current model day. Once again, to eliminate unrealistically-small pig groups, a minimum farrowing size as a proportion of the farrowing farm's total capacity is required for the farrowing function to proceed. Thus, a farm which is already almost at maximum capacity will not farrow a new batch of piglets until another batch has been shipped to an appropriate trading partner.

If the user selects the "All In All Out" biosecurity measure on the setup screen, the above functions will be modified as follows: Instead of a minimum transfer quantity and minimum farrowing quantity, the producer agents will not accept or farrow any new livestock until all stock have been transferred away, and the producer's inventory is at zero.

```
**Recurrence time is the frequency of weaning global parameter**
IF industry role is a farrowing type AND NOT (all-in-all-out AND (non-sow
      inventory > 0))
   // calculate number of farrowed piglets ready to wean and batch
   Current piglet inventory = MIN of remaining pig capacity and (days since last
         weaning day * number of sows * (global parameter for piglets weaned
         annually / 365))
   IF infectivity state is "infected"
      DECREMENT current piglet inventory according to suckling mortality rate
            global parameter
   // wean and batch piglets
   Number to wean and batch = MIN of current piglet inventory and remaining pig
         capacity
   IF (number to wean and batch >= my minimum batch size)
         ADD number to wean and batch and birthday (current day – 35) to pig
               batch tracker
         INCREMENT non-sow pig inventory by batch size
         DECREMENT piglet inventory by batch size
         SET last weaning day to current day
```

Evaluate pig shipments function:

Producers transfer hoofstock to a transferee farm or auction house as soon as the hoofstock reach the age corresponding to the transfer condition associated with the industry role of the producer. If it is determined that the transfer age requirement of a pig batch has been met, a random draw using the auction sales probability determines whether the batch will be sent to auction or directly to producer trading partners.

If it is determined that the animals will be transferred directly, the transferee producers in the transferring producer's pool of possible producer trading partners are sequentially evaluated to determine whether they are able to receive the shipment. To eliminate the transfer of unrealistically-small groups of livestock, transfers will only proceed if the pig batch size exceeds the minimum transfer quantity, as a proportion of the transferee's total capacity. If the capacity of a potential transferee producer is less than the size of the pig batch, the pig batch will be split such that the transferree producer's capacity will be filled. The transferring producer will continue to assess producers until all remaining pigs in the pig batch have been transferred to appropriate trading partners.

If the transferring producer is infected subclinical but the transferee is not infected, the transferred hoofstock will automatically spread the infection to the transferee producer. If the transferee producer is infected but the transferring producer is not, the "delivery trailer" returning from the infected transferee producer may infect the transferring producer according to a probability set at model initialization.

In direct transfers, the birthday parameter associated with the batch of transferred stock is maintained as it is passed to the transferee(s), such that the pig batch will once again be appropriately transferred

to the next production phase at the correct transfer age. The batch is split and delivered to different transferees if there is no one producer that can take the whole batch.

If the transferring producer is infected symptomatic the batch is removed from the system assuming that infected pigs would never be transferred to other premises to avoid spread the infection.

Finishing producers (Feeder to Finish and Farrow to Finish types) ship hoofstock to their slaughter plant, as soon as the hoofstock reach the designated slaughtering age. If the transferring producer is infected, the receiving area of the slaughter plant may become contaminated according to a probability set at model initialization. If the receiving area of the slaughter plant is already contaminated, the “delivery trailer” returning to the transferring producer may carry the infection back to that producer according to another probability set at model initialization.

If the user selects the “All In All Out” biosecurity measure on the setup screen, the above functions will be modified as follows: Instead of a minimum transfer quantity and minimum farrowing quantity, the producer agents will not accept or farrow any new livestock until all stock have been transferred away, and the producer’s inventory is at zero.

```
**Recurrence time is the maximum frequency of pig shipments global parameter**
// to eliminate continually changing inventory levels during execution
SUSPEND farrow, wean, and batch piglets function countdown

// pigs shipped to another producer (referred to as “transferee”)
ELSE
   FOR EACH pig batch meeting age transfer requirement
      FOR EACH transferee in my transferee producers
         IF (batch size <= transferee’s spare non-sow capacity) AND (batch size
                >= transferee’s minimum batch size) AND NOT (transferee uses all-
                in-all-out AND (transferee’s non-sow inventory > 0))
            IF (transferee’s infectivity state is “infected”) AND (infectivity
                   state is “clean”)
               DECREMENT batch size according to mortality rate global parameter
                     associated with pigs’ age
            REMOVE pig batch from pig batch tracker
            DECREMENT non-sow pig inventory by batch size
            ADD pig batch and birthday to transferee’s pig batch tracker
            INCREMENT transferee’s non-sow pig inventory by batch size

            // update contact network trackers
            ADD OR INCREMENT transferee in contact network out-degree list
            ADD OR INCREMENT self in transferee’s contact network in-degree list

            // update pig shipment trackers
            ADD batch size to pig shipments out list
            ADD batch size to transferee’s pig shipments in list
```

```
            // infection brought to transferee via infected pigs
            IF infectivity state is “infected subclinic”
               SET transferee’s infectivity state to “infected”
               ADD OR INCREMENT transferee in infection-spreading network out
                     degree list

            // infection brought home via trailer from transferee farm
            IF (transferee’s infectivity state is “infected”) AND (RANDOM DRAW
                  using Prob. pig truck will become contaminated if producer is
                  infected) AND (RANDOM DRAW using Prob. producer will become
                  infected if returning pig truck is contaminated)
               SET infectivity state to “infected”
               ADD OR INCREMENT self in transferee’s infection-spreading network
                     out degree list

IF industry role is a finishing type
   // pigs shipped to slaughter plant
   ELSE
      REMOVE pig batch from pig batch tracker
      DECREMENT non-sow pig inventory by batch size

      // update contact network trackers
      ADD OR INCREMENT slaughter plant in contact network out-degree list
      ADD OR INCREMENT self in slaughter plant’s contact network in-degree list

      // update pig shipment trackers
      ADD batch size to pig shipments out list
      ADD batch size to slaughter plant’s pig shipments in list

      // infection brought to slaughter plant via infected pigs
      IF (infectivity state is “infected”) AND (RANDOM DRAW using Prob. slaughter
            plant receiving area will become infected if pig batch is infected)
         SET slaughter plant’s infectivity state to “infected”
         ADD OR INCREMENT slaughter plant in infection-spreading network out
               degree list

      // infection brought home via trailer from slaughter plant
      IF (slaughter plant’s infectivity state is “infected”) AND (RANDOM DRAW
            using Prob. pig truck will become contaminated if receiving area is
            infected) AND (RANDOM DRAW using Prob. producer will become infected
            if returning pig truck is contaminated)
         SET infectivity state to “infected”
         ADD OR INCREMENT self in slaughter plant’s infection-spreading network
               out degree list
```

```
IF non-sow pig inventory is 0 SET infectivity state to “clean”
RESUME farrow, wean, and batch piglets function countdown
```

- Feed mill agent cyclically-executing functions

Feed mills periodically generate delivery routes encompassing a subset of producers within their latent feed-mill-to-producer link set. Each route encompasses a subset of the producers in the feed mill’s service area, with the number of stops in each trip resulting from a draw from a Poisson distribution. While there is no actual “feed truck” object in the model, the logic of the following function is based on the way such a truck would move between agents and possibly spread disease.

Beginning from the mill, this conceptual feed truck will visit the previously-drawn number of randomly-selected producers within the feed mill’s service area before finally returning to the feed mill. If the feed mill is infected, the truck may be contaminated initially. Should the truck encounter an infected producer on its route, it may become contaminated at that point. Once a truck is contaminated, the infection may be spread to subsequent producers on the route. If a contaminated truck returns to the feed mill, the mill itself may become infected.

Distribute feed function:

```
**Recurrence time is the frequency of feed deliveries global parameter**
// determine whether truck is initially infected
IF infectivity state is “infected” AND RANDOM DRAW using Prob. feed truck will
      become contaminated if feed mill is infected
   SET truck infected to “true”
ELSE
   SET truck infected to “false”

// determine number of farms on delivery route
SET number to visit to MIN of (number of farms in service area) and (DRAW from
      Poisson distribution with lambda equal to global parameter encoding average
      number of producers visited per route)

// generate delivery route
FOR number to visit
   ADD random producer in service area (that is not already in delivery route
         list) to delivery route list

// parse infectivity consequences of delivery route
FOR EACH producer in delivery route list
// update contact network trackers
   ADD OR INCREMENT producer in contact network out-degree list
   ADD OR INCREMENT self in producer’s contact network in-degree list
```

```
//infected truck infects farm it's delivering to
IF (truck infected is true) AND (RANDOM DRAW using Prob. producer will become
      infected if feed truck is contaminated parameter associated with
      producer receiving the delivery)
   SET producer’s infectivity state to “infected”
   ADD OR INCREMENT producer in infection-spreading network out degree list

//truck becomes infected from delivery to infected farm
IF (producer’s infectivity state is “infected”) AND (RANDOM DRAW using Prob.
      feed truck will become contaminated if producer is infected parameter
      associated with producer receiving the delivery)
   SET truck infected to “true”
   SET “truck-infecting producer” to current producer

// infected truck infects feed mill
IF (truck infected is “true”) AND (RANDOM DRAW using Prob. feed mill will become
      infected if returning feed truck is contaminated)
   SET infectivity state to “infected”
   ADD OR INCREMENT self in truck-infecting producer’s infection-spreading
         network out degree list
```

- Initial infection function

The system is initialized with all agents free of infection. After one model year has passed, an infection is introduced to a random subset of producer agents. The proportion of agents which are infected by the initial infection function may be set at model initialization.

The reason for the one-year lag is to skip the transient period and allow the model to stabilize before analyzing the effect of an introduced disease. This lag is necessary because, as in a real production chain, a certain amount of slack, or a difference between the theoretical production capacity and actual production, is characteristic in the modeled production chains. In the model, this economic slack is due to the producers sometimes temporarily operating at less than maximum hoofstock capacity until an appropriate shipment of livestock becomes available. In general, after about 9 months, the level of slack in the model has stabilized.

Initial infection function:

```
**Function is called only once, after one model year**
FOR number to infect global parameter
   SET randomly chosen producer agent’s infectivity state to “infected”
```

Livestock mortality calculation:

```
DECREMENT piglet inventory by global parameter encoding piglet mortality
      proportion
FOR EACH pig batch in pig batch tracker
   DECREMENT pig batch size by global parameter encoding mortality proportion
         appropriate for age of pigs
```

- Infection control functions

Susceptible/infective state charts:

Each agent has an embedded state chart which encodes its infectivity status (Figure 7). Should an agent become infected, a function is called which calculates the number of its stock which are to die of the disease. The proportion of livestock which succumb to the disease is based on the age of the pig groups, with uniform mortality rates set at model initialization for suckling pigs, nursery pigs, and grow/finish hogs. After die-off is calculated for pig groups of each life stage within an infected producer's inventory, the producer's inventory data are updated accordingly. An agent will remain infected for a duration whose mean length in days is controlled by parameters specific to each agent type.

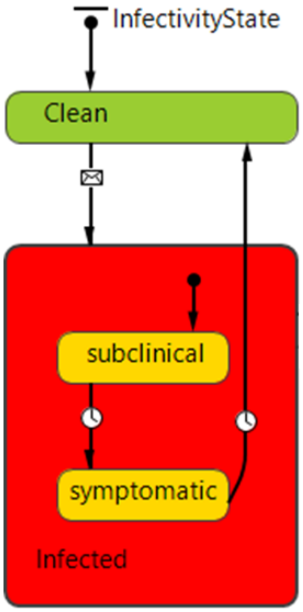

Figure 7: InfectivityState state chart defining disease states and transitions for hog producer agents. Each agent starts in the "Clean" state (no disease) and can transition to the Infected state, which is composite state consisting of subclinical (asymptomatic) and symptomatic sub-states. Transitions are regulated by messages and timeout conditions (two days between subclinical and symptomatic, 50 days from Infected symptomatic to Clean – susceptible). The feed mill and slaughter plant agents can only be clean or infected.

- Risk attitude and biosecurity functions

Both the agent risk attitude and the information provided by the veterinarian agent to the producer agents about the presence of disease regulate the producer's behavior in the transitions between risk-free and risk-present situations. Producer agents can choose whether or not to invest in biosecurity based on disease status updates (number of infected premises in the network) sent by their veterinarian agent (Figure 8). Specifically, each agent's probability of investing in additional biosecurity is governed by a logistic function (Figure 4) and depends on both the number of infected farms within its veterinarian network and the agent's risk attitude. This heuristics are based on information collected by SEGS showing that as infections on nearby farms increase, managers will become more likely to invest in biosecurity, even if it means taking a financial hit up front.

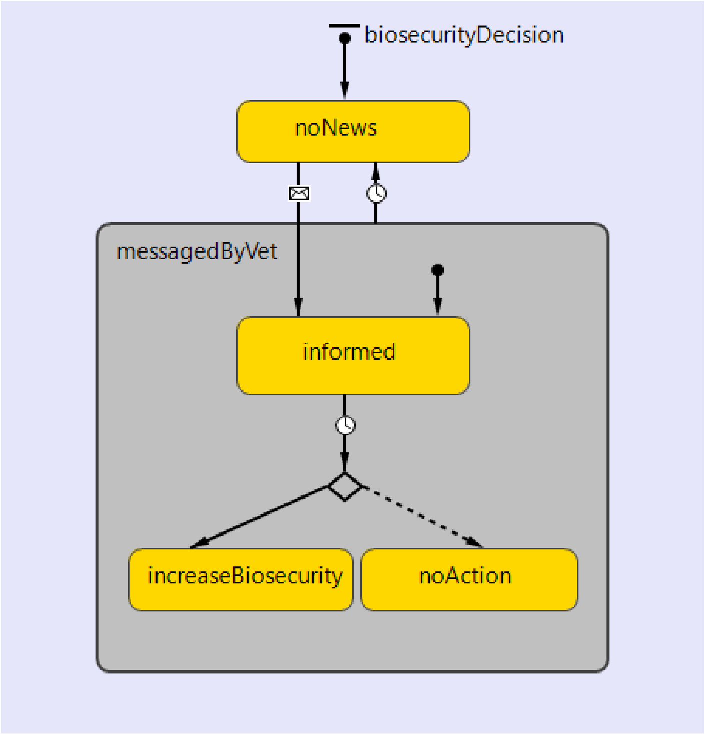

Figure 8: State chart depicting the decision stages of biosecurity investment for a hog producer agent. Messages from the veterinarian on disease status triggers decisions on whether to invest or not. The probability of investment depends on both the message content and the producer's risk attitude.

III.iv.b What are the model parameters, their dimensions and reference values?

See parameter tables above.

III.iv.c How were the submodels designed or chosen, and how were they parameterized and then tested?

The submodels were chosen with the idea to have epidemiological, behavioral and market processes. The largest uncertainty was in the human behavioral parameters. Therefore we used available data to calibrate those parameters and assumed the values of the other submodel parameters obtained with expert knowledge as reasonable and justifiable. The parameterization of the epidemiological submodel was based on expert knowledge from veterinarians. The parameterization of the human behavioral submodels was done with a calibration experiment based on disease incidence observations and data from experimental games.

Due to the inherent variability of epidemic events within complex networked systems, we are interested less in empirically-validating the model to be used as a forecasting tool, and more in developing sufficient structural and face validity to allow for a deeper understanding of the dynamics of the modeled systems. Even given identical starting conditions, deviations in contact patterns over the course of a real-world disease incursion render precise forecasts unfeasible. For example, while reducing outcome volatility in the ABM could easily be accomplished by eliminating stochasticities associated with disease transmission, calibrating the model such that outcomes correspond precisely to a single observed epidemic event misses the point. Our aim is rather to uncover and better understand the fundamental network features that lead to epidemiological vulnerability in livestock production systems more generally.

Calibration and validation procedures that leverage concrete historical data are often regarded as the best way to bring a model in line with empirical evidence. Unfortunately, there is a marked lack of publicly-available data in the agricultural sector beyond aggregated county- or state-level statistics. To the extent that datasets containing explicit locations, operational parameters, livestock and feed movements, and disease histories exist; these data tend to be held by private enterprises, which view them as sensitive internal records.

The spatial locations and basic operational parameters of model agents associated with each study area are calibrated using the ``indirect'' approach, whereby stylized facts about the distribution of agents in the system are gleaned from statistical datasets. Statistical datasets used in this process include the FLAPS output data, USDA data, and livestock and feed movement records we obtained from a large U.S. family-owned hog production chain system (discussed below).

To calibrate additional model elements that define how and when inter-agent contact occurs, as well as epidemiological submodel parameters, we convened several Delphi panels consisting of livestock industry and veterinary experts at national research team meetings and livestock veterinary conferences. In these meetings, we used both qualitative focus groups as well as questionnaires to elicit and hone parameter values. Using this participatory methodology, the modeled system was brought in line with the collective understandings of stakeholders who are intimately familiar with the operational details of U.S. livestock production systems. As model development progressed, these same experts also provided input to ensure the face validity of the distribution of epidemic patterns, scales, and durations produced by the model.

The model's parameters and functions controlling pig movement and feed deliveries were further validated with the help of data provided by a large U.S. family-owned hog production chain system (as a result of our confidentiality agreement, the company's name is not disclosed here). The database

contains two-year records of each pig movement and each feed delivery involving producers in the system, although spatial data on premises locations were not provided. The family-owned hog production chain system consists of a network of 161 producer partners that raise pigs from birth to market. This production chain system has two characteristics that allowed for the parameterization and validation of the ABM. The first characteristic is that the farm sizes vary from small (300 pigs) to large (8800 pigs, Figure 9). The second characteristic is that pigs are grown at specialized sites including farrowing, wean-to-finish, nursery, and finishing premises; and are moved across the production network according to their growing stage. The pig movement records were used to derive realistic estimates of hog transfer frequencies and number of hogs per transfer relative to farm size, including both producer to producer transfers, and producer to slaughter plant transfers (Table 14). The feed delivery records were used to estimate delivery frequencies.

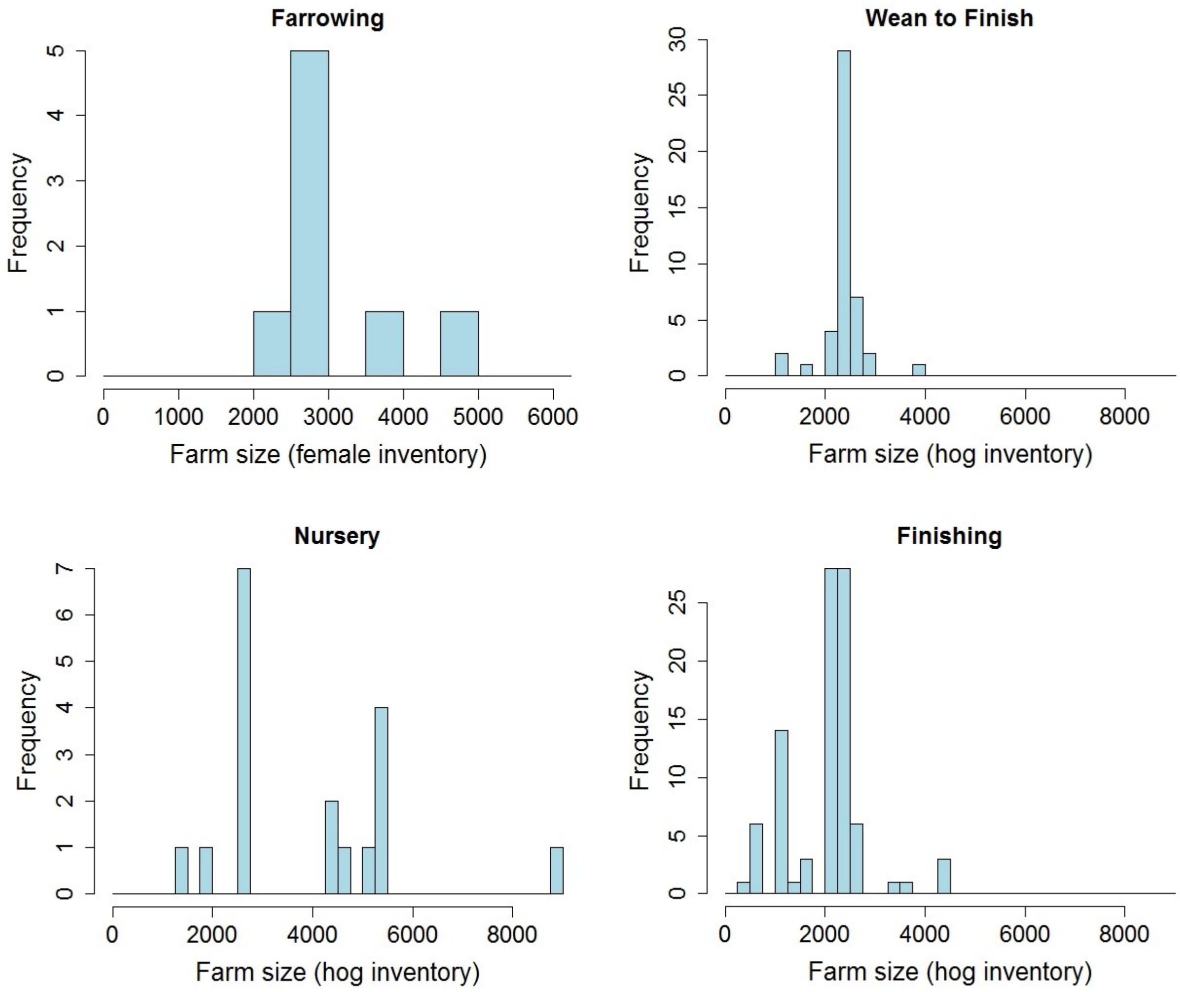

Figure 9: Farm size distribution by farm type (farrowing, wean-to-finish, nursery, finishing)

Table 14: Average annual number of pig shipments within the study period by operational classification

FROM	TO	N shipments FROM	N shipments TO	Avg. shipment size

Wean to Finish	Wean to Finish	1.9	1.6	259
Wean to Finish	Finishing	2.5	1.7	608
Finishing	Finishing	0.5	0.5	176
Nursery	Nursery	1.2	0.8	161
Nursery	Wean to Finish	1.5	1.5	652
Nursery	Finishing	45.7	8.6	512
Supplier	Nursery	156.6	41.2	582
Supplier	Wean to Finish	60.9	10.8	406
Nursery	Customer	0.8	4	3
Finishing	Packer / Customer	27.9	276.6	153
Wean to Finish	Packer / Customer	22.4	179.5	153
Nursery	Packer / Customer	0.5	1	2

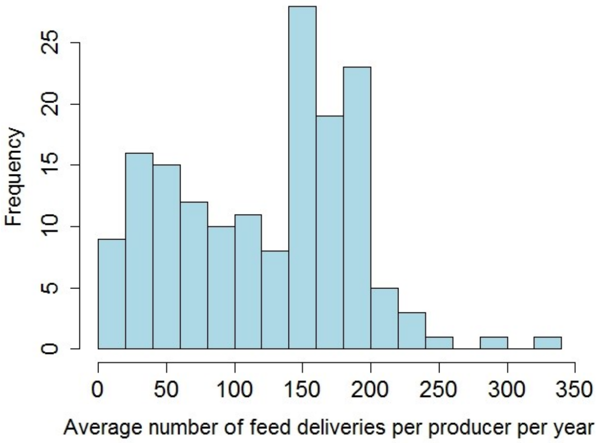

Figure 10: Average number of feed deliveries per producer per year

- Calibration analysis

We calibrated the ABM by matching the weekly incidence from the modelled system and the observed reality, so that the simulation can be closer to the actual case. The analysis was performed in the AnyLogic software environment using a built-in OptQuest genetic algorithm to minimize the Root Mean Squared Error (RMSE) between the modelled and observed incidence over the historical period 05/31/2014 to 02/25/2018. By calibration, we determined four model parameters which control human behavior and about which we lacked data: the increase of biosecurity for each investment, the rate of biosecurity decline due to lack of compliance (psychological distancing), relative difference in the number of infected agents necessary to trigger a biosecurity investment for the three risk attitude groups, and the minimum time lapse allowed between biosecurity investments. We proceeded by a first

calibration with larger ranges of search and then narrowed those ranges around the optimized parameter values for a more refined calibration. Each calibration scenario was run with 100 replicates.

The calibration of the ABM optimized all four parameters with a minimum RMSE of 3.275 in incidence units (Table 15). This means that modelled incidence matched with ≤ 14% error the observed incidence, which ranges between 0 and 23 weekly new infection cases. The parameter combination was carefully cross-checked by running several calibration experiments with different starting parameter values and ranges. The search process yielded other possible parameter assignments with values close to the selected ones but higher RMSE. The calibrated model reproduces the seasonal variability and the negative trend of the observed data but on average, it misses the high winter peaks in incidence (Figure 11).

Table 15: Model calibration results: optimized values for four parameter values linked to human behavior. The last row reports calibration the root mean square error (RMSE) for the optimized parameter combination.

**Calibration parameter**	**Best parameter combination**
Biosecurity increase at investment time	0.3
Biosecurity investment – min. time interval (days)	28
Relative shift of investment probability across risk groups	0.07
Biosecurity compliance – decrease rate	-0.001
Objective value based on RMSE metric	3.275

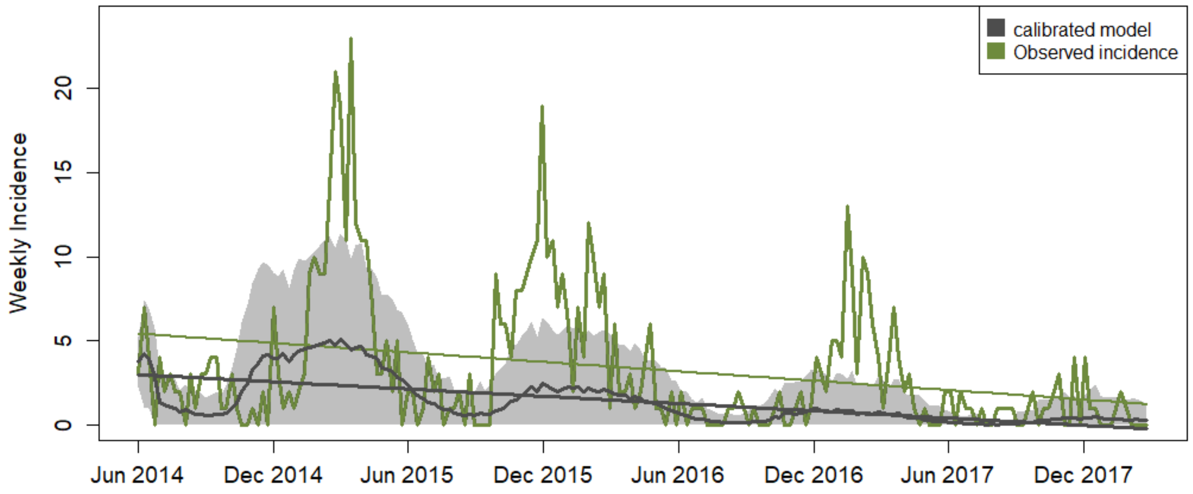

Figure 11: Weekly time series of disease incidence. Data related to the calibrated model outputs are shown in black: average modeled disease incidence (non-linear black line), its standard deviation model-generated across 100 iterations (grey shades) and the linear trend with an average slope of -0.017 ± 0.017. The green line depicts the observed incidence data for North Carolina with a linear trend's superimposed of slope -0.021.

- Sensitivity analysis

Same relative or absolute uncertainty in the different parameters may have hugely different effect on outcomes. Help identify parms/initial states that strongly affect key model results. We place more emphasis in parameter estimation & interventions into parameters exhibiting high sensitivity.

Finally, we conducted a sensitivity analysis focusing on four key parameters. With the sensitivity analysis, we wished to understand the model behavior in response to the variation of four model parameters directly related to disease incidence (Table 16). We use the calibrated model as reference and test conditional variability in incidence by varying one parameter at a time keeping the others at baseline values. No interactions between parameters were tested.

Table 16: Variables used to test model sensitivity. In italic bold are the values of the baseline model obtained from the calibration and used as reference for the analysis. Variables were varied one at a time keeping all the others at the baseline value. Each table cell represents therefore a sensitivity scenario run independently with 500 replicates.

**Parameter**	**Tested parameter values**				
Biosecurity increase at investment time	0.01	***0.3***	0.6	0.9	
Biosecurity compliance – decrease rate (Δ biosecurity/week)	0	***-0.001***	-0.005	-0.01	
Disease subclinical incubation time (days)	0	**2**	7	14	21
Initial infections: Hog Farm; Feed Mill; Slaughter Plant Infections	*3; 1; 0*	15; 5; 0	30; 10; 0	60; 20; 0	

The graphs in Figure 12 display the sensitivity-analysis outputs and can be read as follows. The incidence distribution for the baseline model parameterization can be considered as reference and the other incidence distributions as outputs from different parameterization scenarios. We observe that changes in incidence are significant and follow the directions expected in the real-world observations. Only in two cases (Figure 12, B and C) a change from the baseline to lower parameter values leads to the correct direction of incidence change, but this change is not significant. In the case of biosecurity compliance (Figure 12, B), this means that removing the mechanism of psychological distancing (compliance decrease rate = 0) will not significantly change incidence totals compared to those obtained from the

baseline model with a compliance decrease rate = -0.001. However, more negative decreased rates of compliance do have significant increasing effects on incidence. In the case of disease subclinical incubation time, two versus zero days does not significantly influence the total incidence when the model is initialized with the baseline parameterization (Figure 12 C). However, incubation times ≥ 7 days lead to significant increase in incidence. In summary, we confirmed that the disease mechanisms built in the model and controlled by these four parameters follow realistic behaviors and we can trust the underlying model mechanisms controlled by these parameters.

<table>
<tr><th>Model parameter</th><th>Total disease incidence</th></tr>
<tr><td>Biosecurity increase at investment time with scenario values of 0, 0.3, 0.6, and 0.9 from left to right.</td><td>A

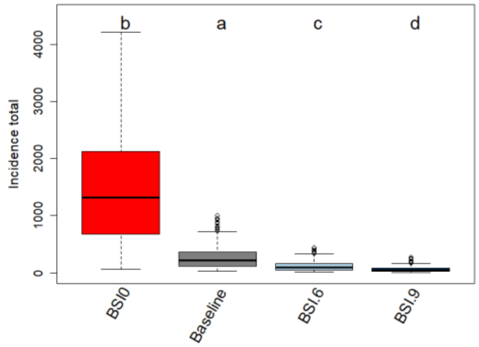

</td></tr>
<tr><td>Biosecurity compliance – decrease rate with scenario values of 0, -0.001, -0.00, -0.01from left to right.</td><td>B

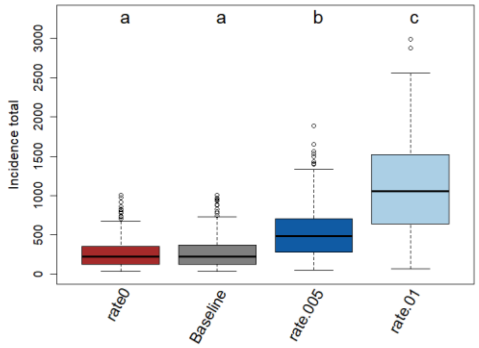

</td></tr>
<tr><td>Disease incubation time measured in days with scenario values of 0, 2, 7, 14, 21from left to right.</td><td>C

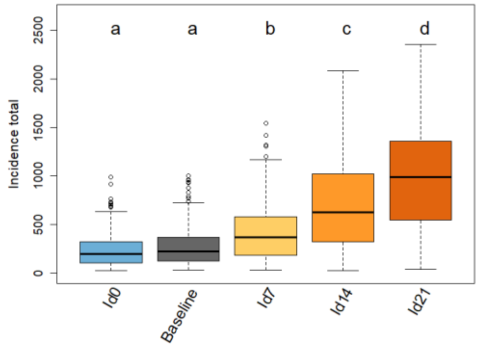

</td></tr>
</table>

Initial number of infections in the three agent populations: hog production premises, feed mills and slaughter plants with scenario values of (3; 1; 0), (15; 5; 0), (30; 10; 0), (60; 20; 0) from left to right.	**D** 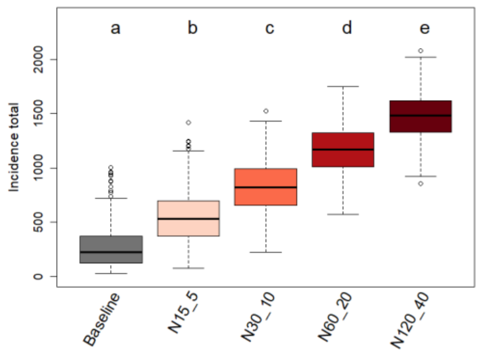

Figure 12: Results from sensitivity analysis. Each table row represents a sensitivity experiment where one model parameter (column 1) was varied across a range of values (scenarios). Box plots of the distributions of disease incidence totals (right column) display comparisons across scenarios where baseline is compared against each scenario. Letters report results from pairwise distribution comparisons of baseline against each scenario. Distributions not sharing any letter are different by the two-sample Kolmogorov-Smirnov test at the 5% level of significance.

Bibliography

Clark, E.M., Merrill, S.C., Trinity, L., Bucini, G., Cheney, N., Langle-Chimal, O., et al. (2020). Using experimental gaming simulations to elicit risk mitigation behavioral strategies for agricultural disease management. *PloS one* 15(3)**,** e0228983. doi: 10.1371/journal.pone.0228983.
